# The relationship between sternum variation and mode of locomotion in birds

**DOI:** 10.1186/s12915-021-01105-1

**Published:** 2021-08-19

**Authors:** Talia M. Lowi-Merri, Roger B. J. Benson, Santiago Claramunt, David C. Evans

**Affiliations:** 1 Department of Ecology and Evolutionary Biology, University of Toronto, 25 Willcocks Street, Toronto, ON M5S 3B2 Canada; 2 Department of Natural History, Royal Ontario Museum, 100 Queen’s Park, Toronto, ON M5S 2C6 Canada; 3 Department of Earth Sciences, University of Oxford, South Parks Road, Oxford, OX1 3AN UK

**Keywords:** Ecomorphology, Geometric morphometrics, Sternum, Avian, Flight

## Abstract

**Background:**

The origin of powered avian flight was a locomotor innovation that expanded the ecological potential of maniraptoran dinosaurs, leading to remarkable variation in modern birds (Neornithes). The avian sternum is the anchor for the major flight muscles and, despite varying widely in morphology, has not been extensively studied from evolutionary or functional perspectives. We quantify sternal variation across a broad phylogenetic scope of birds using 3D geometric morphometrics methods. Using this comprehensive dataset, we apply phylogenetically informed regression approaches to test hypotheses of sternum size allometry and the correlation of sternal shape with both size and locomotory capabilities, including flightlessness and the highly varying flight and swimming styles of Neornithes.

**Results:**

We find evidence for isometry of sternal size relative to body mass and document significant allometry of sternal shape alongside important correlations with locomotory capability, reflecting the effects of both body shape and musculoskeletal variation. Among these, we show that a large sternum with a deep or cranially projected sternal keel is necessary for powered flight in modern birds, that deeper sternal keels are correlated with slower but stronger flight, robust caudal sternal borders are associated with faster flapping styles, and that narrower sterna are associated with running abilities. Correlations between shape and locomotion are significant but show weak explanatory power, indicating that although sternal shape is broadly associated with locomotory ecology, other unexplored factors are also important.

**Conclusions:**

These results display the ecological importance of the avian sternum for flight and locomotion by providing a novel understanding of sternum form and function in Neornithes. Our study lays the groundwork for estimating the locomotory abilities of paravian dinosaurs, the ancestors to Neornithes, by highlighting the importance of this critical element for avian flight, and will be useful for future work on the origin of flight along the dinosaur-bird lineage.

**Supplementary Information:**

The online version contains supplementary material available at 10.1186/s12915-021-01105-1.

## Background

The transition from terrestrial theropod dinosaurs into volant modern birds (Neornithes) presents one of the most well-documented major transitions in evolutionary history [1–6]. The ability to locomote using powered flight, in which the wings generate lift and propulsive force, is a major innovation that expanded the ecological opportunities of early birds and contributed to their extant diversity [7, 8]. Neornithes is the most diverse group of terrestrial vertebrates today, with over 10,000 named species that occupy a broad range of morphological and ecological niches across the globe. They also engage in a diverse range of locomotory strategies, with flight styles that include continuous flapping, soaring, and burst flight, and other locomotory modes, such as foot-propelled diving, wing-propelled diving, and running in flightless birds [9–11]. Despite a significant body of literature on the transition from ground-dwelling dinosaurs into birds, there are still large gaps in our understanding of how and when powered flight originated, and how it has evolved since its origin.

The sternum, or breastbone, sits in the middle front portion of the ribcage in birds and is present in either ossified or cartilaginous form in most terrestrial vertebrates. Most flying neornithine birds possess a median bony projection, the keel, that projects ventrally along the midline of their sternum, serving as the anchor for major flight muscles that insert on the wing (e.g., *m. pectoralis* and *m. supracoracoideus*) [12]. A single ossified sternum featuring a prominent keel has been regarded as a necessity for powered flight numerous times over the last century, however without much quantitative support [2, 4, 12–18]. Outside of birds, extinct flying reptiles (pterosaurs) employ strong powered flight and have a single ossified sternum with a small, cranially projecting spike which resembles some greatly reduced version of a keel [19], while flying mammals (bats) have a very narrow midline sternum that bears a low median ridge [20]. Although some form of a keel may be necessary for powered flight in general, the uniquely enlarged and morphologically varied sternum in Neornithes implies greater importance of the element to anchor flight muscles in avian flight.

**Fig. 1:**
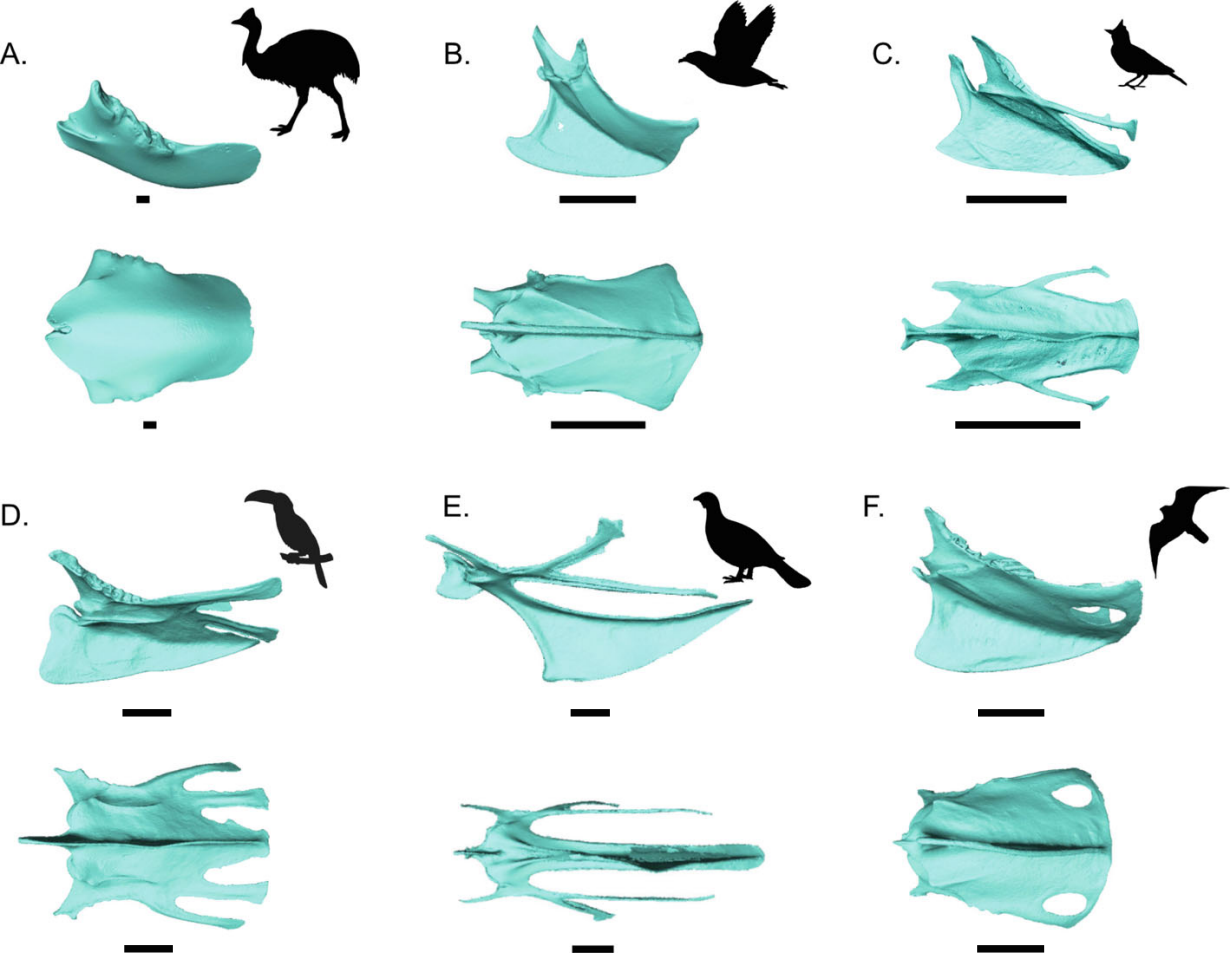
Overview of sternal variation. Sterna shown in lateral view (above) and ventral view (below). **A** *Casuarius casuarius* (Southern cassowary); **B** Oceanodroma leucorhoa (Leach’s storm petrel); **C** Calandrella cinerea (Red-capped lark); **D** Ramphastos ambiguous (Yellow-throated toucan); **E** Alectoris chukar (Chukar); **F** Falco sparverius (American kestrel). Scale bars: 10 mm. Silhouettes sourced from phylopic.org, credited to (in order of appearance): Casuarius casuarius uncredited; Procellariifomes by Juan Carlos Jerí; Aluadidae uncredited; Ramphastidae by FJDegrange; Phasianidae by Elisabeth Östman; Falco by Liftarn

Avian sternum morphology varies considerably in a myriad of traits, including relative size, overall shape, number of trabeculae or fenestrae, and keel height and curvature (Fig. 1), which is likely related to the varying levels of mechanical strain imparted by the pectoral and oblique muscles used in flight, or in locomotion more generally [12, 21]. While a strong functional relationship has been established between flight ability and wing shape [22–25], sternal form-function relationships are more poorly understood due to a lack of detailed studies. Elementary inferences of a relationship between sternal shape and flight mode go back to the nineteenth century, when researchers noted that raptorial birds typically have fenestrae on the caudal ends of their sterna, diving birds have long, narrow sterna, and flightless birds lack keels [12, 15]. While early interpretations like these have become fixed through decades of literature [3, 26–29], these ecomorphological hypotheses have still not been adequately tested, save for a few descriptive and preliminary morphometric studies [30–36]. Sternum ecomorphology remains a critically unexplored component to understanding the evolution and origins of powered flight.

The relationship between flight and sternum shape requires more detailed study, and considering recent debates over the origin of powered avian flight [5, 37–40], a rigorous analysis of sternal ecomorphology is particularly timely. The aim of this study is to provide the first large-scale, quantitative comparative analysis of neornithine sternum variation. Here, we quantify sternum morphology across a broad phylogenetic scope of neornithine birds and identify morphological features that are associated with locomotory modes using geometric morphometrics and phylogenetic comparative methods. We explicitly test the allometric relationship between sternum size, sternum shape, and body mass, as well as a number of foundational ecomorphological hypotheses of the relationships between sternum shape and locomotory capabilities. We hypothesize that sternum size and shape will be directly related to muscular forces exerted onto various parts of the sternum related to flight modes, or to locomotor-related variation in body shape that influences the trunk profile. Previous studies have demonstrated that overall, flight muscles scale isometrically with body mass [41–43], and therefore we predict that sternum size will remain proportional to body mass. We also predict that sternum shape will differ between locomotory modes and independently of phylogeny, that flightless birds will have reduced or absent sternal keels, and that flight styles requiring high flapping power will be associated with sterna that have greater ossified surface area. The results of testing these key hypotheses relating sternal variation to flight and locomotor mode will provide important evolutionary insights into form-function relationships in Neornithes, and lay the groundwork for understanding the origin and evolution of powered flight in the avian stem lineages.

## Results

**Fig. 2:**
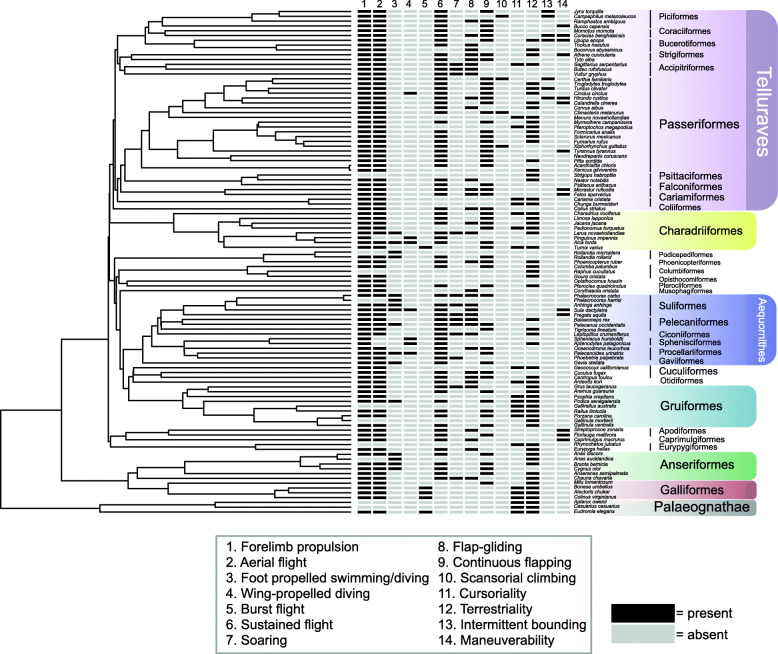
Phylogenetic tree showing all 105 taxa used in dataset. Locomotory categories are shown in legend, and classifications of “present” and “absent” are coded in a data matrix in black and grey, respectively. Clade names are provided on the right side of the data matrix

We performed a 3D geometric morphometric analysis to examine relationships between sternum size, sternum shape, locomotion, body size, and phylogeny. To do this, we collected morphometric data using landmarks and semi-landmarks on 3D surface scans of 105 neornithine sterna across a broad phylogenetic range and showing various locomotory abilities (Fig. 2). First, we report on the results of some widely used morphometric approaches: (1) assessment of sternum size allometry, and (2) principal component analysis of sternal shape variation. We then report (3) a formal statistical analysis of the relationship between sternum shape variation, size variables, and locomotory ability using a distance-based phylogenetic generalized least squares (pGLS). Variables describing locomotory ability are defined in Table 1. We employed various model selection approaches, in which different combinations of explanatory variables (size and locomotory variables) were tested against sternum size or sternum shape to obtain the model which best fit the shape data (see “Methods”).

**Table 1:** Descriptions of the locomotory modes included in this study; descriptions compiled from published sources [9, 10, 44–46]

Locomotory mode	Description
*Forelimb propulsion*	Use of forelimbs to produce lift and/or propulsion. This includes all birds that fly, as well as wing-propelled divers, such as penguins and auks. This is to avoid the simple dichotomy of “flighted” and “flightless” birds, as wing-propelled diving requires strong pectoral muscles. Species coded as 0 in this category would be flightless birds that do not engage in wing-propelled diving or flight.
*Aerial flight*	Flight in air regardless of lifestyle (flightless wing-propelled divers are excluded).
*Sustained flight*	Flight that can be sustained over medium to long distances.
*Terrestriality*	Terrestrial locomotion and lifestyle (terrestrial, coded as 'present') as opposed to an alternative lifestyle (i.e., aquatic, arboreal; coded as 'absent'). Although this category does not explicitly pertain to locomotion, it is relevant such that a terrestrial lifestyle influences the manner in which birds takeoff and land, which may influence both flight style and sternum shape.
*Cursoriality*	Running as a frequent form of locomotion.
*Burst flight*	Flight that involves explosive burst of flapping in takeoff to escape predator. Seen in Galliformes, Tinamiformes, and some Charadriiformes. This category is often conflated with short escape flights of birds with poor flight capabilities; however, the distinction between the two is crucial, as this specific burst flight behavior is not present in all poorly flying birds and may influence sternal morphology.
*Wing-propelled diving*	Locomotion involving swimming or diving underwater using primarily forelimb propulsion. This includes flightless wing-propelled divers, such as penguins and the great auk, as well as flying birds that can also dive using wing propulsion, such as dippers, diving petrels, and boobies.
*Foot-propelled swimming and diving*	Locomotion involving swimming or diving underwater using primarily hindlimb propulsion. This includes surface swimmers, like ducks, and underwater divers like loons and grebes. Some birds in this category are also wing-propelled divers.
*Continuous flapping*	Type of flight that primarily involves flapping continuously without interspersed gliding or soaring.
*Flap-gliding*	Flight that involves bursts of wingbeats that are interspersed with short periods of gliding (sometimes referred to as “undulating flight”). This does not refer to glides used when landing.
*Soaring*	Flight involving gliding either using thermal updrafts (“thermal soaring”), ridge lifts, or wind gradients (“dynamic soaring”).
*Scansoriality*	Locomotion involving climbing tree trunks and branches.
*Intermittent bounding*	Flight that involves bursts of wingbeats interspersed with short periods with the wings folded against the body.
*Maneuverability*	Flight that is highly maneuverable with fast and frequent aerial turns.

### Sternum size allometry and locomotory correlates

**Fig. 3:**
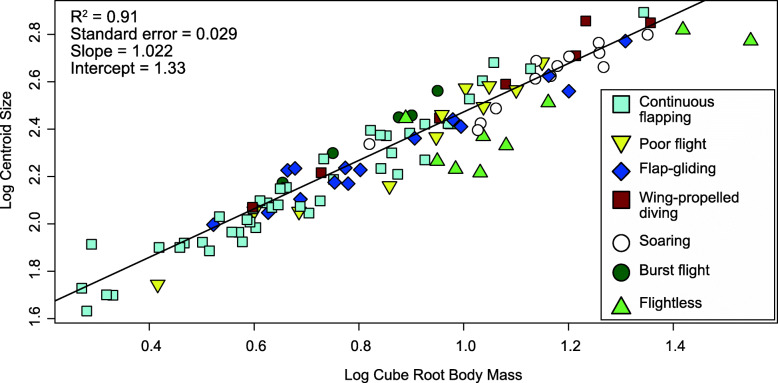
Allometry of sternum centroid size against body size. Log-transformed cube-root body mass is plotted against log-transformed centroid size, with each point representing an individual specimen. The solid line is the regression line corresponding to the presence of forelimb propulsion in the pGLS model *size ~ body mass + forelimb propulsion*. The intercept shown on this plot incorporates the coefficient value for forelimb propulsion in the model. Datapoint colours and symbols indicate the predominant locomotory mode of each species (legend provided). Test statistics provided in Table 2

To test for allometry in sternum size, we compared pGLS models that explain sternum centroid size (the dependent variable), using combinations of body mass and various locomotory variables (Table 1). Model selection using the corrected Akaike Information Criterion (AICc) favored the following model: *sternal size ~ body mass + forelimb-propulsion* (AICc weight = 0.72; Fig. 3; Table 2). This indicates that larger birds have bigger sterna, and that forelimb-propulsion has independent statistically significant influence on residual variation in sternal size after accounting for body mass. The slope of body mass in this model is indistinguishable from isometry (slope = 1.0222; S.E. = 0.0292; Table 2), and forelimb propulsion has a coefficient of 0.123 (S.E. = 0.0201, Table 2). This indicates that birds of similar body masses and that use forelimb propulsion (either subaqueous, aerial or both) have an estimated 0.123 log_10_ units larger sterna than those incapable of forelimb propulsion, or a 33% increase in sternal size associated with forelimb propulsion.

**Table 2:** Regression statistics for all the size allometry regression models with AICc weights > 4.0 × 10^−6^, testing for allometry of sternum size. BM = body mass, size = sternum centroid size, FP = forelimb propulsion, AF = aerial flight, BF = burst flight, SO = soar, FPD = foot-propelled swimming/diving, WPD = wing-propelled diving. *N* = 105 for all analyses

Models	AICc	AICc weights	***R***^**2**^	Pagel’s λ	Variable	Coefficient	Std. error	***t***-value	***p*** value
**size ~ BM + FP**	− 243.9	0.72	0.91	0.78	Intercept BM FP	1.33 1.022 0.12	0.049 0.029 0.0201	27.32 35.02 6.13	0 0 0
size ~ BM * FP	− 240.6	0.14	0.91	0.78	Intercept BM FP BM:FP	1.46 0.903 − 0.013 0.13	0.12 0.098 0.11 0.102	12.70 9.18 0.12 1.26	0 0 0.91 0.21
**size ~ BM + FP + BF**	− 240.55	0.14	0.91	0.75	Intercept BM FP BF	1.31 1.029 0.123 0.074	0.048 0.029 0.020 0.039	27.038 35.56 6.067 1.89	0 0 0 0.062
**size ~ BM + FP + AF + FPD**	− 234.35	6.1 × 10^−3^	0.91	0.80	Intercept BM FP AF FPD	1.35 1.003 0.21 − 0.093 0.027	0.049 0.030 0.045 0.042 0.023	27.27 33.36 4.73 − 2.22 1.203	0 0 0 0.029 0.23
**size ~ BM + FP + AF + BF + SO**	− 229.52	5.4 × 10^−4^	0.90	0.79	intercept BM FP AF BF SO	1.33 1.015 0.19 − 0.075 0.072 − 0.006	0.052 0.032 0.043 0.042 0.039 0.023	25.704 31.26 4.47 − 1.79 1.84 − 0.29	0 0 0 0.076 0.068 0.77
**size ~ BM + AF**	− 228.58	3.4 × 10^−4^	0.90	0.75	Intercept BM AF	1.35 1.027 0.086	0.053 0.032 0.020	25.65 31.75 4.25	0 0 0
**size ~ BM + FP + AF + FPD + WPD**	− 227.35	1.8 × 10^−**4**^	0.90	0.80	Intercept BM FP AF FPD WPD	1.35 1.003 0.20 − 0.079 0.023 0.017	0.050 0.030 0.053 0.050 0.024 0.033	27.18 33.22 3.73 − 1.59 0.97 0.52	0 0 0.0003 0.12 0.34 0.60
**size ~ BM + FP + AF + FPD + WPD + BF**	− 224.51	4.4 × 10^−5^	0.90	0.76	Intercept BM FP AF FPD WPD BF	1.32 1.012 0.20 − 0.079 0.029 0.017 0.080	0.049 0.030 0.053 0.049 0.023 0.032 0.039	27.032 33.99 3.75 − 1.59 1.25 0.53 2.073	0 0 0.0003 0.12 0.21 0.60 0.041
**size ~ BM + FP + AF + CF + BF + SO**	− 221.14	8.3 × 10^−6^	0.90	0.78	Intercept BM FP AF CF BF SO	1.32 1.022 0.19 − 0.079 0.0092 0.077 − 0.0035	0.053 0.034 0.044 0.043 0.016 0.0401 0.024	25.084 29.75 4.40 − 1.84 0.56 1.92 − 0.15	0 0 0 0.068 0.57 0.057 0.88
**size ~ BM**	− 219.9	4.5 × 10^−6^	0.89	0.73	Intercept BM	1.46 0.983	0.049 0.0328	30.11 29.936	0 0

Plotting the relationship between sternum size and body mass reveals unique relationships of locomotor traits with sternal size, combined with the non-random distribution of locomotor traits with respect to body mass (Fig. 3). For example, some flight styles are restricted to one section of the plot, indicating that certain flight styles only occur above certain size classes. Birds that weigh below ~ 37 grams (g) are only either continuous flappers or poor fliers (Fig. 3). The smallest wing-propelled diver in the dataset, *Cinclus cinclus*, weighs 62 g, and the smallest soaring bird, *Larus novaehollandiae*, weighs 289 g. Continuous flappers and flap-gliders have predominantly smaller body sizes, while soarers and flightless birds are predominantly larger (Fig. 3).

A model containing an interaction between body mass and forelimb propulsion (sternal size ~ body mass * forelimb propulsion) was less well-supported (AICc weight = 0.14; Table 2). This indicates that the presence or absence of forelimb propulsion does not influence the slope of the relationship between sternal size and body mass. The model containing *sternum size ~ body mass + forelimb propulsion + burst flight* showed equivalent AICc support as the previous model (AICc weight = 0.14), with the “burst flight” variable showing marginal non-significance (*p* = 0.062; Table 2). This suggests that although it is currently not statistically supported, there may be a possible effect of burst flight ability being correlated with sternum size. A simpler model of *sternum size ~ body mass* shows much poorer AICc support (AICc weight = 4.5 × 10^-6^; Table 2).

**Fig. 4:**
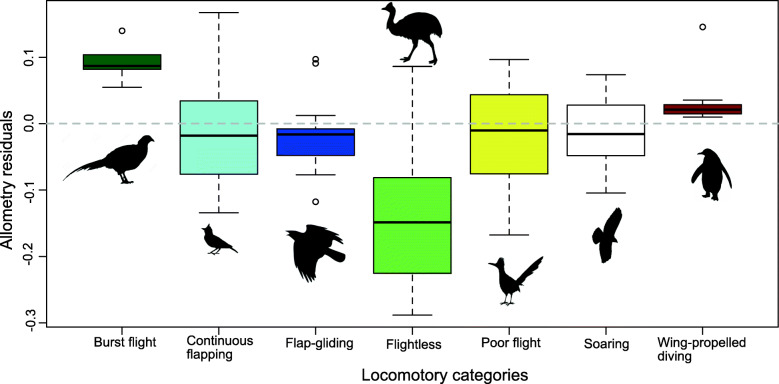
Residuals from Fig. 3 plotted against simplified locomotory categories. The regression line, or zero on the y-axis, is indicated by the dotted grey line. Black lines indicate the median, boxes indicate the first and third quartiles, and whiskers and open circles indicate minimum and maximum values for each locomotory category. Silhouettes of birds that typically use this locomotory mode are included; silhouettes are not to scale. Silhouettes sourced from phylopic.org, credited to (from left to right): Phasianidae by Mattia Menchetti; Aluadidae uncredited; *Corvus* by Peileppe; *Casuarius casuarius* uncredited; *Geococcyx* by Patrick Fisher (vectorized by T. Michael Keesey); *Buteo* by Lauren Anderson; *Aptenodytes* by Steven Traver

Independent effects of the simplified locomotor variables are shown in Fig. 4 as the residual values from the best pGLS model (*sternal size ~ body mass + forelimb propulsion*). Sternum size relative to body size is much smaller in flightless birds as indicated by these residuals, and burst fliers have larger relative sternum size to body mass than the other groups (Fig. 4). Regression statistics and AICc scores and weights for the remaining allometry models that were tested are provided in Additional file 1: Table S1.

### Principal component analysis

**Fig. 5:**
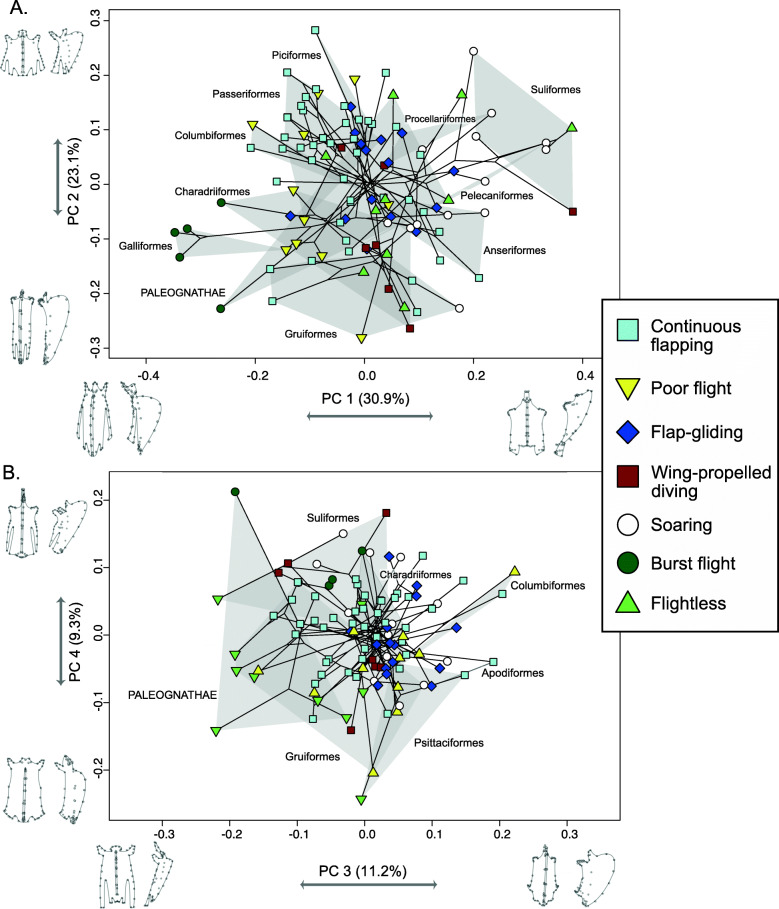
Morphospaces showing **A** PC1 and PC2 axes and **B** PC3 and PC4 axes plotted against each other, with sternum shape warps associated with the extremes of each principal component indicated along each axis in ventral view (left) and lateral view (right). Individual point color and symbol indicate the predominant simplified locomotory mode of each species (legend provided). Major taxonomic groups that separate out at the periphery of the morphospace are shown in gray polygons. **A** PC1 and PC2 axes shown. **B** PC3 and PC4 axes shown. Phylogeny is overlain on top of the data points

We performed a principal component analysis (PCA) to visualize the shape variation in sternal morphology. The first four principal components (PCs) of sternal shape variation accounted for 74.6% of the cumulative shape variance (Fig. 5). Subsequent axes each accounted for < 5% of the shape variance, and thus will not be discussed. PC1 (30.9%) describes variation in sternal elongation. Negative values of PC1 are assigned to elongate sterna that expand caudally with long, rounded lateral trabeculae, whereas positive values of PC1 are assigned to shorter, more square-shaped sterna with shallow and cranially projecting keels, one wide lateral trabecula on each side, and a concave caudal edge (Fig. 5A). PC2 (23.1%) also represents sternal elongation, without the concave caudal edge seen in PC1 (Fig. 5A). Negative values of PC2 are assigned to narrow, elongated sterna with rounded keels, while positive values of PC2 are assigned to shorter sterna with one lateral and one intermediate trabecula on each side, and narrow craniolateral processes. All burst-flying taxa show negative values for both PC1 and PC2. Positive PC1 values show mostly soaring birds, with other modes overlapping with soaring at lower positive PC1 values. These other locomotory modes overlap near the center of the plot. The phylogeny mapped onto the morphospace shows the tree branches scattered around the plot, with locomotory groups plotting close together in morphospace despite coming from distantly related taxonomic groups (Fig. 5A). This indicates that there is considerable convergent evolution in sternum morphology.

The next two PC axes, PC3 and PC4, also show substantial overlap in morphospace among most locomotory categories (Fig. 5B). PC3 (11.2%) shows variation in keel size and caudal border morphology. Negative values of PC3 are assigned to rectangular sterna with straight lateral edges, shallow keels, and long lateral trabeculae that produce a concave caudal edge, while positive values of PC3 are assigned to sterna with prominent, rounded keels, rounded caudal borders, and shorter craniolateral processes. PC4 (9.3%) is primarily driven by the angle of the keel. Negative PC4 values are assigned to sterna with shorter, rounded keels, and positive PC4 values are assigned to sterna with taller, acutely angled, and cranially projecting keels. Flightless birds appear to cluster together, having negative values for both PC3 and PC4, while burst flying birds cluster together with negative PC3 values and positive PC4 values. The positive end of PC3 is occupied only by flying birds (specifically continuous flappers, flap-gliders, poor fliers, and soarers). Like the previous PCA plot, there is substantial phylogenetic convergence in both sternum morphology and locomotory mode. Results from the analysis of shape allometry based on PC scores are provided in Additional file 1, Figure S1, and Table S2.

### Analysis of sternal shape variation

#### Model selection

To test the relationships between sternum shape, sternum size, body mass, and various locomotory variables, we compared various Procrustes-distance pGLS models containing these variables (see “Methods”). Taxa were assigned a value of “present” or “absent” per locomotory variable to account for multiple locomotory styles being present within a species (Fig. 2). Model selection procedures favored a comprehensive model formulated as *sternal shape ~ body mass + sternum size + forelimb-driven lift-based propulsion + aerial flight + terrestriality + cursoriality + burst flight + foot-propelled swimming* and *diving + continuous flapping + soaring* (Table 3). This model indicates that there are significant correlations between body mass, sternum size, and all the included locomotory traits with sternum shape while controlling for shared phylogenetic history (*p* < 0.01). The independent effects of these variables on sternal shape derived from this model are discussed below. The remaining locomotory variables (wing-propelled diving, flap-gliding, scansoriality, intermittent bounding, maneuverability) were not found to have a significant influence on sternal shape in this analysis and are not discussed further.

**Table 3:** ProcD.pgls ANOVA table for the selected best fit model to the sternal shape, indicating the variables that are most significantly correlated with shape. df = 1 for all variables

	SS	MS	***R***^**2**^	***F***	***Z***	***p*** value
Sternum size	0.0074	0.0074	0.053	12.44	6.01	0.001 **
Body mass	0.0052	0.0052	0.038	8.80	5.42	0.001 **
Soaring	0.0044	0.0044	0.032	7.45	4.55	0.001 **
Continuous flapping	0.0034	0.0034	0.025	5.78	4.21	0.001 **
Terrestriality	0.0033	0.0033	0.024	5.57	3.93	0.001 **
Burst flight	0.0032	0.0032	0.023	5.32	3.65	0.001 **
Cursoriality	0.0029	0.0029	0.021	4.90	4.14	0.001 **
Foot-propelled swimming and diving	0.0026	0.0026	0.018	4.30	3.95	0.001 **
Forelimb propulsion	0.0023	0.0023	0.017	3.89	3.49	0.001 **
Aerial flight	0.0018	0.0018	0.013	3.11	2.90	0.002 **

#### Allometry of sternal shape

**Fig. 6:**
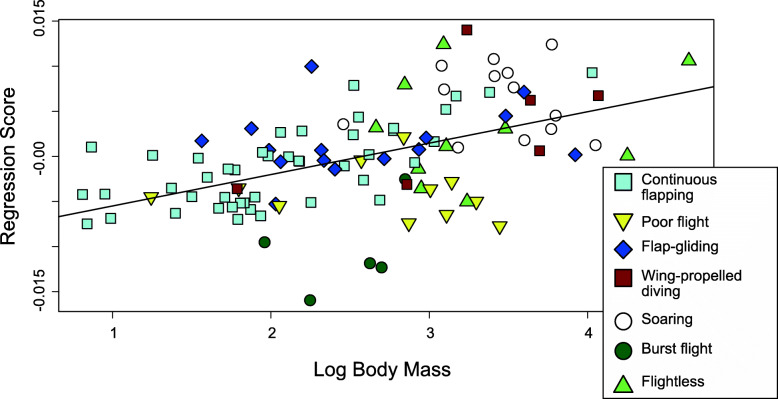
Overall sternal shape, represented by the Regression Score, plotted against log-transformed body mass, with each point representing an individual specimen. The solid line indicates the regression line from the best procD.pgls model *sternal shape ~ body mass + sternum size + forelimb-driven lift-based propulsion + aerial flight + terrestriality + cursoriality + burst flight + foot-propelled swimming and diving + continuous flapping + soaring* (slope = 0.00354, S.E. = 0.000578, *p* = 1.67 × 10^−8,^ *R*^2^ = 0.267). Datapoint colors and symbols are indicated by simplified locomotory categories provide in the legend

**Fig. 7:**
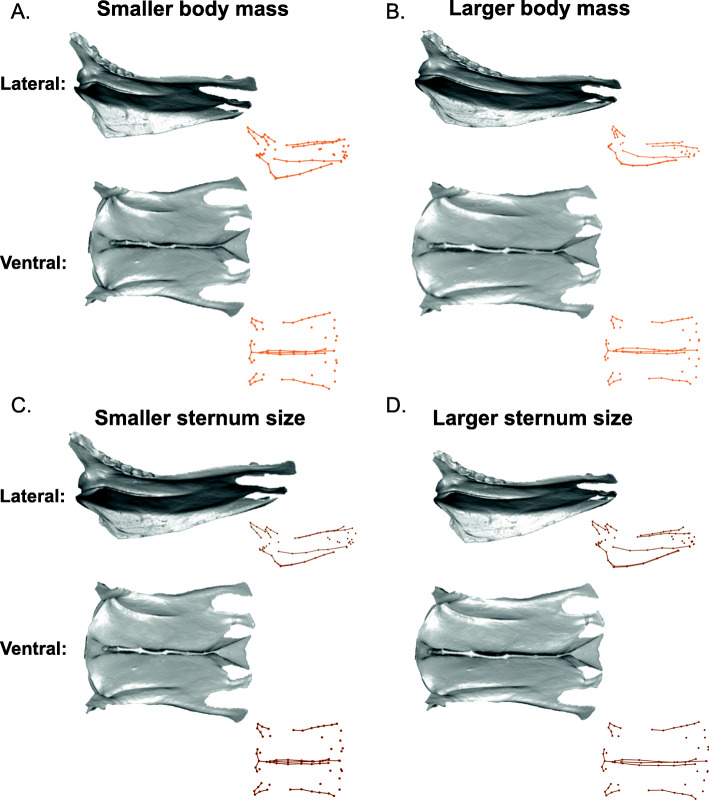
Sternal shape deformations showing the independent effects of sternum size and body size on sternum shape, with “smaller” indicating the first quartile of size, and “larger” indicating the third quartile of size. 3D models in grey are warped from the *Ramphastos ambiguus* sternum according to the algorithm indicated by the point- and line-clouds to the bottom-right of each 3D model. 3D models in grey are warped from the *Ramphastos ambiguus* sternum according to the algorithm indicated by the point- and line-clouds in the bottom right corner of the 3D models

The best model indicates that body mass is significantly correlated with sternal shape (Table 3). The relationship between these two variables is visualized in Fig. 6, in which body mass is plotted against the regression score of the model, which represents overall shape. Shape deformations associated with variation in body mass and sternum centroid size were plotted in 3D shape space to assess specific morphological associations with size (Fig. 6, Additional file 1: Fig. S2). Both body mass (*R*^2^ = 0.038, *Z* = 5.3, *p* = 0.001) and sternum size (*R*^2^ = 0.0532, *Z* = 6.0463, *p* = 0.001) have a significant relationship with sternal shape. The smaller body mass (first quartile) is associated with a mediolaterally wider sternum and slightly taller keel, while larger body mass (third quartile) is associated with a narrower sternum and a lower, rounded keel (Fig. 7A, B, Additional file 1: Fig. S2). The effect of sternum size is similar to body mass, except that the larger sternum size is associated with a relatively taller sternal keel (Fig. 7C, D, Additional file 1: Fig. S2). These results indicate that smaller birds (with smaller body masses) tend to have broader sterna, and larger birds have narrower sterna with proportionally lower keels, independent of sternum size and locomotory mode. Smaller sterna (with smaller sternal centroid sizes) are also proportionally broader with lower keels, while larger sterna are narrower; however, larger sterna are associated with relatively taller keels independent of body size and locomotory mode.

#### Sternal shape and locomotion

**Fig. 8:**
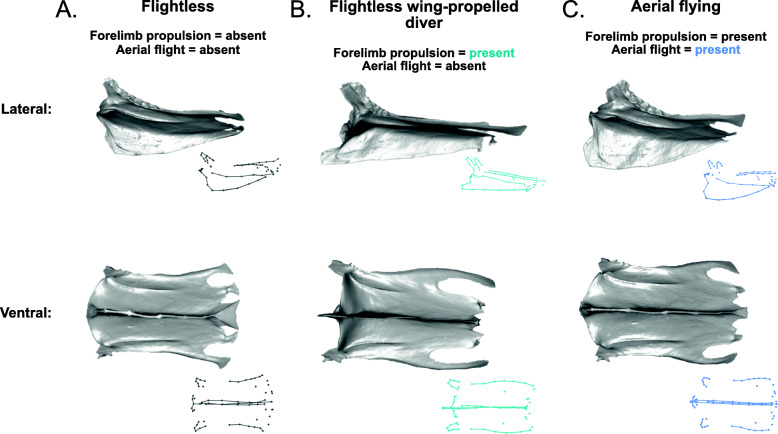
Sternal shape deformations showing the independent effects of forelimb propulsion and aerial flight. 3D models in grey are warped from the *Ramphastos ambiguus* sternum according to the algorithm indicated by the point- and line-clouds to the bottom-right of each 3D model. Forelimb propulsion is associated with a longer sternal body and shallower, cranially projected keel; aerial flight is associated with a shorter sternal body, a deeper, rounded keel, and rounded lateral trabeculae.

A completely ‘flightless’ sternum, which shows the average shape for sterna that code as ‘absent’ for all locomotory variables in the model, is rectangular in shape, slightly expanded caudally, and has a shallow rounded keel (Fig. 8A, Additional file 1: Fig. S3). Forelimb propulsion has a significant relationship with sternal shape in the model (*R*^2^ = 0.017, *Z* = 3.5, *p* = 0.001). The independent effect of forelimb propulsion involves a shallower, acutely cranially projecting keel and more elongated, rounded lateral trabeculae, compared with birds that are not capable of forelimb propulsion (either subaqueous or aerial; Fig. 8B, Additional file 1: Fig. S3). The independent effect of aerial flight (*R*^2^ = 0.013, *Z* = 2.9, *p* = 0.002) shows a shorter sternum and a taller, rounder keel (Fig. 8C, Additional file 1: Fig. S3).

**Fig. 9:**
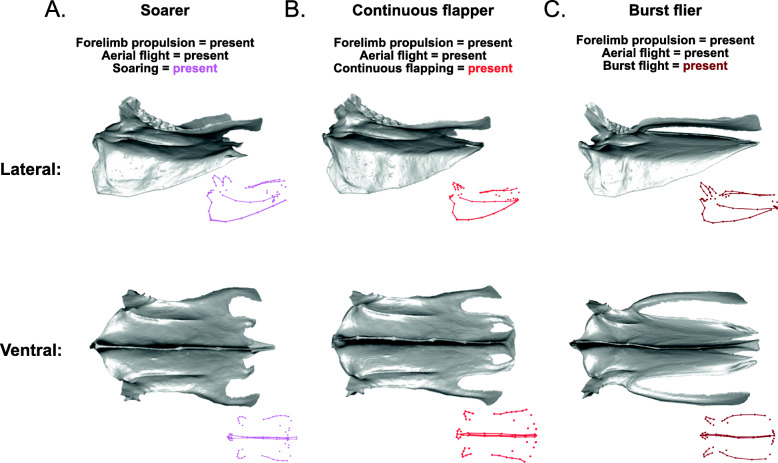
Shape deformations showing the independent effects of soaring flight, continuous flapping flight, and burst flight. 3D models in grey are warped from the *Ramphastos ambiguus* sternum according to the algorithm indicated by the point- and line-clouds to the bottom-right of each 3D model

**Fig. 10:**
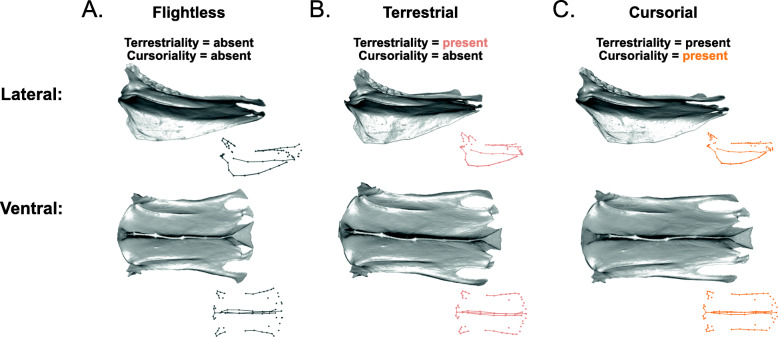
Shape deformations showing the independent effects of terrestriality and cursoriality. 3D models in grey are warped from the *Ramphastos ambiguus* sternum according to the algorithm indicated by the point- and line-clouds to the bottom-right of each 3D model

**Fig. 11:**
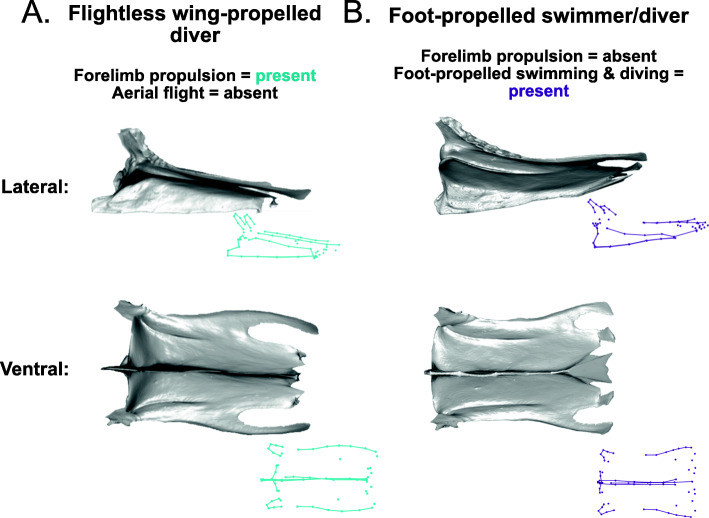
Shape deformations showing the independent effects of **A** forelimb propulsion, provided for comparison with **B** foot-propelled swimming and diving. 3D models in grey are warped from the *Ramphastos ambiguus* sternum according to the algorithm indicated by the point- and line-clouds to the bottom-right of each 3D model

Soaring flight (*R*^2^ = 0.032, *Z* = 4.6, *p* = 0.001) results in sterna that are narrower in the middle and expand caudally, have rounded craniolateral processes, a cranially projecting keel, shorter intermediate trabeculae than lateral trabeculae, and a xiphoid process that bears the caudal end of the keel (Fig. 9A, Additional file 1: Fig. S4). Continuous flapping (*R*^2^ = 0.025, *Z* = 4.2, *p* = 0.001) produces deeper sternal notches, distally expanded lateral and intermediate trabeculae, and a round keel that does not project as far cranially (Fig. 9B, Additional file 1: Fig. S4). Burst flight (*R*^2^ = 0.023, Z = 3.7, *p* = 0.001) results in very deep distal sternal notches, long, narrow lateral trabeculae, and narrower craniolateral processes (Fig. 9C, Additional file 1: Fig. S4). Terrestriality (*R*^2^ = 0.024, *Z* = 3.9, *p* = 0.001) shows a shape deformation with a narrow sternal body, and a taller keel, compared with flightless sterna lacking terrestriality (Fig. 10A, B, Additional file 1: Fig. S5). Cursoriality (*R*^2^ = 0.021, *Z* = 4.1, *p* = 0.001) results in an even more narrow and elongated sternal body, and a rounder keel that is angled more caudally (Fig. 10C, Additional file 1: Fig. S5). Finally, foot-propelled swimming and diving (*R*^2^ = 0.018, *Z* = 3.9, *p* = 0.001) results in sterna that are medially narrow and caudally expanded and have a shallower but acutely cranially projecting keel (Fig. 11B, Additional file 1: Fig. S6).

## Discussion

This study presents the first large-scale ecomorphological assessment of the relationship between sternal shape, size, and locomotion in Neornithes. The ecomorphological significance of the sternum has been overlooked in most influential studies of avian flight [11, 37, 47–50], despite its fundamental importance. Moreover, understanding of sternal ecomorphology has important potential to clarify the evolution of powered flight in the fossil record. Our analyses suggest that the relationship between sternal shape and locomotion is complex, with some aspects of sternal morphology showing stronger correlations with locomotor ability than others, while a substantial portion of sternal shape variation remains unexplained. Flying birds always have prominent sternal keels, and meaningful differences between sternum shape exist between various locomotory modes, including soaring, burst flight, continuous flapping, and cursoriality.

### Allometric and locomotory effects on sternal size

We found that sternum size is nearly isometric with body size in Neornithes. This is consistent with some previous studies that demonstrated an isometric relationship between pectoral muscle mass and body mass [41–43]. In contrast, Neornithes show positive allometry for wing size, with disproportionately larger wingspans or wing areas in larger birds, as seen also in fossil paravians on the neornithine stem lineage [9, 11, 51–53]. The different relationships of sternal size and wing size with body mass may be explained by the active biomechanical role of wings in flight, compared with the passive role of the sternum in anchoring flight muscles.

We also found that forelimb propulsion accounts for a significant portion of sternum size variation, indicating that birds with forelimb propulsion have ~ 33% larger sterna relative to their body size compared to birds that lack forelimb propulsion, either aerial (flying) or subaqueous (wing-propelled swimmers, including, e.g., penguins) (Figs. 3 and 4). Most birds that lack forelimb propulsion have reduced pectoral musculature, which naturally correlates with smaller sterna and an absent or greatly reduced sternal keel. A few such birds do not have reduced sternum size (namely *Anas aucklandica* and *Raphus cucullatus*), which may be explained by a more recent loss of flight in these taxa. The degree of sternum reduction in these birds may be related to the amount of evolutionary time since flight was lost in the lineage, with *Anas aucklandica* diverging from its closest flighted relative ~ 1 Mya [54]. The divergence time of *Raphus cucullatus* from its closest flighted relative is older, estimated at ~ 18 Mya [55], and the body mass recorded for *Raphus* is an estimate rather than a direct measurement [56], so this case should be interpreted cautiously.

Burst fliers have larger sternum sizes relative to body size (Fig. 4), although this effect is marginally non-significant and receives equivocal AICc support (Table 2). It therefore requires further investigation. However, if supported, then the larger sterna of burst fliers may be explained by their short, powerful flapping bursts that require greater power and thus greater muscle mass and a corresponding larger sternum for muscle attachment [57].

Overall, our analyses of sternum size demonstrate it to be a useful tool for estimating flight abilities of taxa in the fossil record. Apart from lineages that lost flight recently, a sternum size-to-body size ratio can be a relatively reliable indicator of powered, propulsive flight, with larger ratios indicating the presence of powered flight and smaller ratios suggesting the absence of flight (Figs. 3 and 4).

### Sternal shape, ecomorphology, and allometry

Phylogenetic comparative analyses reveal that sternum morphology is significantly influenced by allometry and by various locomotory traits, which collectively explain up to 26.4% of shape variation (Table 3). Explanatory power for each variable is low (Table 3), leaving a substantial portion of unexplained variation in total. Various factors might potentially explain this. For example, locomotion is complex and difficult to capture into discrete categories without the risk of over- or under-parameterization. Our categories are broad and capture first-order locomotor capabilities. However, it was not possible to capture the complexity of all avian behaviors. For example, our classification does not incorporate takeoff and landing abilities, feeding ecology associated with locomotion, dances or flights used in courtship displays, or other life history traits like egg size or nesting behavior. Therefore, we can surmise that unexplained morphological variation in the sternum may be characterized by unidentified functional traits, as well as growth, development, and shared ancestry. Nevertheless, convergent evolution of sternal morphology is evident in some taxa that share specific locomotory abilities (e.g., the similar sternal shapes in burst-fliers such as Tinamiformes, Galliformes, and Turnicidae within Charadriiformes), indicating the potential predictive power of sternal shape for some locomotor traits. Among our locomotory traits, forelimb propulsion and aerial flight both have statistically significant independent effects on sternal morphology, as does soaring, continuous flapping, burst flight, terrestriality, cursoriality, and foot-propelled swimming and diving.

The presence of shape allometry is revealed by both body size and relative sternum size, which have similar but distinct effects on sternal shape. Larger body sizes, while holding relative sternum size constant, is associated with a narrower sternal shape and a shallower keel (Fig. 7B), and larger sternal sizes, while holding body size constant, is also correlated with a narrower sternum and a deeper keel (Fig. 7D). Nudds and Rayner [58] argued that larger birds have relatively wider and flatter body (i.e., trunk) shapes than smaller birds to provide more lift during flight. However, their measurement of skeletal width reflected the distance between the two coracoids, which can vary in positioning on the sternum: several larger-sized birds show coracoid sulci on the sternum that overlap one another, while birds of all sizes show coracoid sulci at variable distances from one another and positioned at various angles (personal observation). Further, sternum width may not directly reflect body width, as muscle mass on the sides of the body may vary. Therefore, considering our results, it is worth exploring the relationship between sternum width and total body width, to determine whether there is a meaningful correlation between body size and streamlining of the body profile [44].

#### Forelimb propulsion, diving, and drag

Flightlessness, swimming, and aerial flight are each characterized by distinct sternal morphologies. The average shape of sterna in taxa that lack forelimb propulsion (i.e., do not use the forelimb for propulsion in either in air or water) is rectangular with a low, rounded keel (e.g., Fig. 8A). The use of wings for underwater propulsion only (i.e., “underwater flight” in flightless wing-propelled divers such as penguins) is associated with an elongated sternum and a distinct, acutely angled and cranially projecting keel (Fig. 8B). In contrast, aerial flight is associated with shorter sterna and much taller, rounder keels (Fig. 8C). Perhaps surprisingly, wing-propelled diving on its own, independent of aerial flight capability, does not exhibit a significant independent effect on sternum shape. This pattern of associations indicates that morphological adaptations for wing-propelled diving are distinctive only in flightless divers. Flying wing-propelled divers instead have sternal shapes more similar to those of other flying birds, likely due to the primacy of functional constraints required for aerial flight [59].

This pattern of sternal shape associations is reflected by other observations of flightless wing-propelled divers. The vastly different densities of air and water are likely associated with different body shapes in aerial fliers compared to subaqueous diving birds [59]. Penguins (Spheniscidae) and the extinct great auk (Alcidae), use their forelimbs exclusively for wing-propelled diving and have substantially reduced pectoral muscles compared to diving birds that are also capable of flight, such as extant auks (Alcidae), diving petrels (Procellariidae), gannets (Sulidae), and dippers (Cinclidae) [59]. They also have highly modified wings, which are substantially flattened and stiffened, have thicker cortical bone walls, and lack an alula digit, all of which aid in streamlining the hydrofoil in wing-propelled diving [59, 60].

The sternal shape of flightless wing-propelled divers such as penguins closely resembles the sternal shape associated with foot-propelled diving in our analysis. Both have shallower, cranially projecting keels compared to other birds (Fig. 11A, B). We suggest this sternum shape allows for a dorsoventrally flatter body cross-section, which may result in a more hydrodynamic, streamlined profile [61–63]. Diving birds typically also have a number of other adaptations for streamlining, including smoother feathers, hindlimbs placed far back on the body, and a laterally compressed tarsus [61, 64]. Therefore, we hypothesize that selective pressures on streamlining of diving bird body shape produce a convergence in sternum shape regardless of the use of the wings from underwater propulsion or not.

#### Soaring flight and flapping speed

Out of all the locomotory modes analyzed, soaring flight had the strongest statistical correlation with shape. Soaring birds have a shorter sternum that narrows in the middle and expands caudally, have a concave caudal margin with one lateral trabecula and a shorter intermediate trabecula on each side, and have a slightly cranially angled keel (Fig. 9A). They typically have longer wings and larger deltopectoral crests compared to flapping birds, which confers greater surface attachment area for the *m. pectoralis*, or the downstroke muscle, on the forelimb [65, 66]. According to lever theory, a longer moment arm can typically exert a greater force, but at a lower angular velocity [67]. By extension, this suggests that the longer deltopectoral crests in soaring birds are more biomechanically suitable for slow but powerful wingbeats, while birds with shorter deltopectoral crests have an advantage for faster flapping. Soaring birds also require very little upstroke power from the *m. supracoracoideus*, as they are supported by aerodynamic and thermal lift rather than requiring an active upstroke [11, 68]. Therefore, we predict that the deeper sternal keel in soaring birds may be needed to accommodate a larger muscle mass, mostly of *m. pectoralis*, for greater downstroke strength needed for moving larger wings.

#### Continuous flapping and ventilation

Our results suggest that more aerobic forms of flight, like continuous flapping, are associated with greater surface area for respiratory muscle attachment onto the sternum, compared with birds that use less aerobic (e.g., burst flight) or less energetic (e.g., soaring) forms of flight. The sternal shape associated with continuous flapping, which was subtle but statistically significant, exhibits two deeper sternal notches on each side, and both lateral and intermediate trabeculae that widen distally and may fuse with the xiphoid process (Fig. 9B). Abdominal and oblique muscles (e.g., *m. obliquus abdominis externus* and *m. rectus abdominis*), whose primary function are in ventilation, attach directly onto the lateral trabeculae and caudal sternal border, respectively [69, 70]. These trunk muscles activate to lower the sternum ventrally, expanding the abdomen and facilitating air flow into the abdominal air sacs during inspiration [71, 72], and subsequently pull the sternum dorsally during expiration [73]. Burst flight, which is primarily anaerobic [74] is associated with an elongated sternal body, narrow lateral trabeculae, and a single deep sternal notch on each side (Fig. 9C), which provides less ossified surface along the trabeculae and caudal sternal border for ventilatory muscle attachment. These are conspicuous morphological features that occur in all three of the burst-flying groups analyzed here (landfowl (Galliformes), tinamous (Tinamidae), and buttonquails (Turnicidae))^1^. Soaring flight, which is the least energetically expensive of these three flight modes, is associated with reduced lateral and intermediate trabeculae, leaving little ossified surface along the caudal border for muscle attachment. Further, continuously flapping birds have higher metabolic rates and thus greater oxygen consumption rates than soaring birds (which can partially rely on air currents for propulsion [9, 74]) and typically increase both ventilation and oxygen consumption rates while in flight [78]. Therefore, it is reasonable to predict that the predominantly oxidative continuous-flapping flight [74] would also require greater muscular action to raise and lower the caudal end of the sternum. Here, we support the hypothesis that possessing a more ossified caudal sternal border, either with expanded trabeculae (as seen in continuous flappers, Fig. 9B), trabeculae fused distally to the xiphoid process forming fenestrae (e.g., *Falco sparverius*, Fig. 1F), or a solid border with no notches or fenestrae (e.g., *Oceanodroma leucorhoa*, Fig. 1B), allows for greater attachment of abdominal muscles involved in ventilation and is thus correlated with highly aerobic flight styles like continuous flapping. While we also see expanded lateral trabeculae in flightless sterna, this is absent in the shape deformations associated with forelimb propulsion and aerial flight alone. Therefore, this hypothesis requires further testing.

#### Terrestriality and cursoriality

The sternal shape deformation associated with terrestriality shows a narrowing of the caudal border (Fig. 10B), which may also signal a proportionally narrower trunk cross-section. This can be explained as a potential way of making room for having larger hindlimb muscles in terrestrial birds and for facilitating forward movement of the hindlimbs [79–81]. Cursorial birds, which require even stronger and more efficient hindlimb musculature for running, appear to have more narrow sterna overall (Fig. 10C), likely for the same reason. Their longer, narrower sternum may also be related to a shift in the center of mass on the bird, with more efficient runners having a center of mass closer to the pelvic region, while less efficient runners and non-running birds have a further cranial center of mass [82]. The caudal shift of the apex of the keel may be explained by the allocation of more muscle mass to the caudal part of the sternum, contributing to a caudal shift of the center of gravity.

### Implications

#### The sternal keel

Our results support previously held notions that the presence of a prominent sternal keel is fundamental for powered flight ability in Neornithes [2, 4, 12–18]. Furthermore, in birds that have a dorsoventrally shallow trunk cross-section (e.g., in subaqueous diving birds), development of a cranially projecting keel may compensate for presence of a ventrally shallower keel, allowing the maintenance of forelimb propulsive ability (Fig. 11). This sort of morphology is also seen in pterosaurs, which have shallow ossified sterna with a cranially projecting spike, similar to some birds [19]. As mentioned above, a taller keel may be correlated with a longer deltopectoral crest on the humerus, suggesting that the height of the keel may have more to do with the power and action of the *m. pectoralis*, the downstroke muscle, than the *m. supracoracoideus*, the upstroke muscle, which lies closer to the body. Previous studies on flight take-off performance in birds have found that downstroke velocity and amplitude are at their maximum during takeoff flight, and taper off during steady flight [83]. Therefore, it would be worthwhile to explore the ecomorphology of takeoff flight in the sternum across the various avian flight styles.

#### Sternum morphology and the origins of avian flight

Most studies on flight in early fossil paravians have focused primarily on feather arrangements and skeletal forelimb anatomy in a relative few prominent fossil species [37, 40, 47, 65, 84–88]. These provide information on various parameters related to flight capabilities, such as wing loading, lift, and drag coefficients [39], but do not generate a complete picture of avian flight ecomorphology. Our study provides the framework to incorporate the morphology of the avian sternum into ecomorphological interpretations of fossil bird locomotion, and by extension, the origin and sequence of acquisition of flight capabilities in the stem lineage more broadly. While our results do not provide direct correlates of specific flight modes in the sternum, we do present two main criteria for identifying the presence of powered flight in Neornithes: (1) the presence of a large sternum relative to body size, and (2) the presence of a deep and/or cranially projected sternal keel (i.e., a proportionally large surface area of the keel). The first potential evidence of a keeled sternum in stem birds is in *Confuciusornis* from the clade Pygostylia. Here, the sternum is flat, with some specimens showing a slight ridge along the midline, which has been interpreted either as a keel homolog or a potential anchor for a cartilaginous keel [89, 90]*.* Further crownward on the avian stem, Enantiornithes exhibit a wide variety of sternal morphotypes which include a ventrally projecting sternal keel, and several enantiornithine birds are thought to have been capable of powered flight [16, 45, 91, 92]. Fossil evidence suggests that the sternal keel is likely to have evolved as early as Pygostylia, and definitely by Euornithes, based on the phylogenetic hypotheses presented by [39]. Extending our conclusions directly to infer flight capabilities in stem birds should be done cautiously. However, they suggest so far that either these early stem taxa had more limited sustained flight capabilities than extant birds, or that their flight-related musculoskeletal function, and therefore sternal morphologies and flight modes, were fundamentally different than they are in modern Neornithes. Future analysis of fossil paravian sterna is needed to illustrate the significance of sternal morphology for understanding the early evolutionary stages of avian flight.

## Conclusions

We present the first study to rigorously analyze the ecomorphology of the avian sternum, despite it being a key element for powered flight. Our morphometric analysis uses a comparison across a set of Procrustes-distance regression models to explicitly test hypotheses and characterize the independent effects of multiple interacting variables on sternal shape using high-dimensional shape data, multi-dimensional locomotor traits, and indices of body size and sternal size. This establishes a more complete and more explicit methodology for studying the effects of allometry and ecology on morphology, which we apply to the avian sternum. Analyses reveal two morphological features that are necessary for powered avian flight: (1) a large relative sternum to body size, and (2) a deep or cranially projected sternal keel (i.e., a proportionally large surface area of the keel). Correlations of specific locomotory modes with shape are significant but show low predictive power, possibly due to the difficulty in capturing the complexity of avian locomotion in our broad locomotory categories. However, distinct morphological changes are evidently associated with different locomotory categories. Deeper keels tend to be correlated with slower but stronger wingbeats, and robust caudal sternal borders provide attachment surfaces for muscles involved in ventilation, which may strengthen under higher-powered flapping, or flight requiring higher metabolic rates. Sternal size and shape are both most strongly influenced by body size and forelimb propulsion ability, with shape also being strongly influenced by sternal size. Certain shapes are notably associated with locomotory modes, specifically the short sternal body and trabeculae associated with soaring, the elongated sternal body and lateral trabeculae associated with burst flight, the expanded trabeculae associated with continuous flapping, and the narrower sternal body found in terrestrially and cursorially adapted birds. Our conclusions also provide a strong basis for future estimations of the potential locomotory capabilities of stem Neornithes based on the size and shape of their sternum, and illuminate the origin and early evolution of powered avian flight. Understanding the significance of the sternum in avian flight fills a substantial gap in knowledge of the relationship between skeletal form and function in one of the most species-rich and ecologically diverse groups of terrestrial vertebrates, and reveals numerous promising new avenues for functional and evolutionary research.

## Methods

We apply a phylogenetically informed method for performing analysis of variance (ANOVA) and regression models on highly multivariate data introduced by Adams [46], which allows for direct hypothesis tests on the relationship between shape and ecology [93–95]. This contrasts with the widespread application of principal component analysis (PCA) in the study of ecomorphology [96–98]. PCA is fundamentally an ordination method that returns the major axes of shape variation. This is useful for visualization purposes, but does not directly evaluate ecomorphological hypotheses or distinguish the independent effects of multiple, potentially interacting variables, and can therefore lead to overreaching ecological interpretations. Further, principal component (PC) axes can only represent few aspects of shape variation at a time, meaning that using PC axes as shape variables in hypothesis tests cannot account for the entire shape at once. We combine both the traditional PCA method along with Adams’ phylogenetic multivariate method (distance-based phylogenetic generalized least squares, or pGLS) [46] to incorporate the sternal shape in its entirety, and to test hypotheses on the relationship between shape and ecology.

### Data acquisition

Three-dimensional computed tomography (CT) and surface scan data of sterna from 105 skeletally mature bird specimens were collected from museum collections. Each specimen in our dataset represents a different species across 62 families. Species were selected to exhibit taxonomic and locomotory breadth, as well as to capture independent evolutionary origins of different locomotory strategies, such as wing-propelled diving, cursoriality, and flightlessness within the orders of Neornithes (Fig. 2). These instances were identified when a species exhibited locomotory traits that were unique among its order, and this species along with a close relative were both included. This design is intended to maximize the statistical power in phylogenetic approaches [99].

Morphological surface data was acquired using one of three methods, depending on resource availability: CT scanning, surface scanning using a handheld Artec Space Spider 3D scanner, or a NextEngine 3D desktop laser scanner. 3D models of the sterna were then constructed using the 3D visualization software Avizo (Thermo Fisher Scientific, Waltham, MA, USA). Our scans and surface models are available on MorphoSource [100]. The body masses for particular specimens were recorded when available; otherwise, we used the species’ average body mass collected from the Dunning Handbook for Avian Body Masses [101]. For the two extinct species in the dataset, the great auk (*Pinguinus impennis*) and the dodo (*Raphus cuculatta*), body mass estimates were included from published sources [56, 102].

### Locomotory categories

We developed a classification of locomotory modes based on previous studies [9, 10, 68] (provided in Table 1). The following locomotory categories are included: forelimb propulsion, aerial flight, foot-propelled swimming and diving, wing-propelled diving, burst flight, sustained flight, soaring, flap-gliding, continuous flapping, terrestriality, cursoriality, scansoriality, intermittent bounding, and maneuverability (explanations provided in Table 1). These different locomotory categories are not mutually exclusive, as some categories are nested within each other (e.g., aerial flight cannot occur without forelimb propulsion), and because a species may be capable of performing more than one of the alternate modes. These locomotory styles include both flight and non-flight categories, as they all convey relevant locomotory data with hypothesized associations to sternal morphology. Categorization of each of these modes for all taxa was accomplished using *Handbook of Birds of the World* [103] and the *Cornell Lab of Ornithology* 'Birds of the World' [104] online databases. Modes were coded as 'present' or 'absent' for each species so that multiple locomotory modes could be assigned. 'Aerial flight' and 'forelimb propulsion' are separated to account for birds that engage in underwater forelimb propulsion for swimming, such as penguins, but are not capable of aerial flight. These birds would code 'present' for forelimb propulsion and 'absent' for aerial flight, while flying birds would code 'present' for both categories. Our multivariate approach to coding ecological trait variation accommodates species that are capable of multiple flight modes, originating with the multivariate classification scheme of [10]. While we recognize that modern avian flight is complex, with multiple variations on flight modes or intermediate abilities, our categories remain broad to avoid having categories with too few species.

### Phylogeny

We controlled for phylogenetic non-independence in several analyses by using a synthetic tree containing 9993 extant avian species (Fig. 2) [105]. Two extinct species absent from the synthetic tree, *Raphus cuculattus* (the dodo) and *Pinguinus impennis* (the great auk), were added next to their sister species using the bind.tip function in the package phytools [106] in R 3.6.2 [107], with branch lengths calculated based on their estimated divergence times [55, 108]. Taxa in the original synthetic tree not included in our 3D sternal morphology dataset were then pruned from the tree prior to analysis. The final tree can be found in our Dryad repository [109].

### Geometric morphometrics

**Fig. 12:**
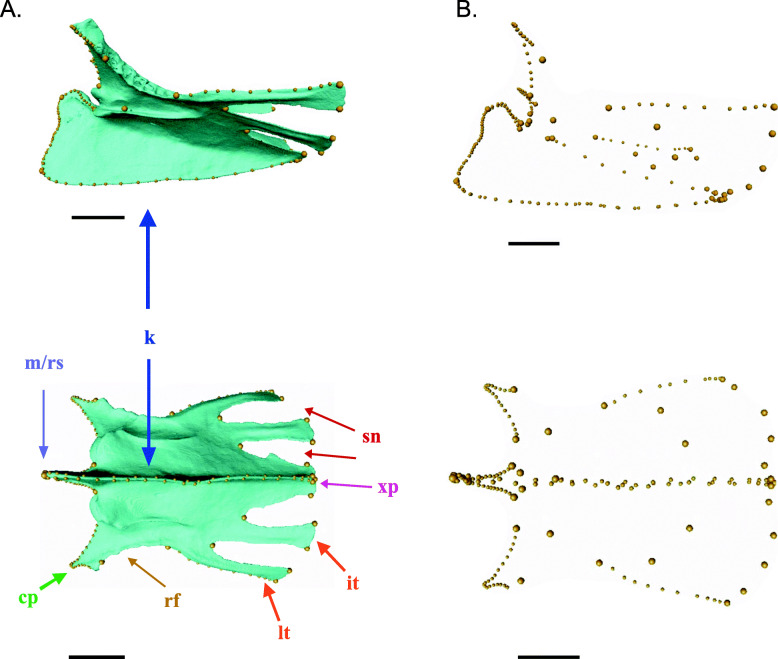
**A** 3D model of sternum from *Ramphastos ambiguus* with anatomical labels showing noteworthy regions on the sternum, with landmarks (larger yellow spheres) and semi-landmarks (smaller yellow spheres) along edges and curves, with the lateral view on top and ventral view below. Abbreviations: cp, craniolateral process; it = intermediate trabeculae; k, keel; lt, lateral trabeculae; m/rs, manubrium/rostrum sterni; rf, rib facets; sn, sternal notches; xp, xiphoid process. **B** Landmarks extracted from the 3D model, with the lateral view on top and ventral view below. More detailed figure of landmarks provided in Additional File 1 Figure S1. Scale bars: 10 mm

A series of 32 landmarks and 9 sets of sliding semi-landmarks were placed along the most variable portions of the sternum body using the visualization software Avizo (Thermo Fisher Scientific, Waltham, MA, USA) (Fig. 12; see Additional File 1 Supplementary Methods, Fig. S1). The number of semi-landmarks in each series varied across specimens during placement, and once the curvature was captured, semi-landmarks were resampled to a set of equally spaced points along each curve, with point counts equal to the lowest number of semi-landmarks initially placed for each semi-landmark series across all specimens. For specimens that were broken on one side, landmarks were reflected along the midline from the opposite side. It should be noted that, while some very minor individual variation in sternal shape exists, the general aspects of shape variation are constant within species. Our landmarks were chosen with the intent to capture the major aspects of sternal morphological variation, including sternal length, width at different points along the sternum, keel height and curvature, sternal notch depth, and number of lateral and intermediate trabeculae.

Landmark configurations were aligned by generalized Procrustes superimposition using the gpagen function in the R package geomorph [110]. This minimizes the bending energy differences among landmark constellations by translating them to a shared centroid position, scaling them to a unit centroid size, and rotating them to minimize the residual distances of landmark coordinates from their mean shape, while allowing semi-landmarks to slide along their tangent vectors [111–114]. The Procrustes coordinates resulting from this procedure are used as a generalized measure of sternal shape. Centroid size, used here as a proxy for sternum size, is calculated as the square root of the summed squared distance of each landmark from the centroid position [111].

#### Analysis of sternum size

We evaluated the relationship between sternum centroid size and body mass through phylogenetic generalized least squares (pGLS) regression [115]. This provides a test of the allometry of sternum size and a base model by which to explore the mass-independent effects of locomotory variables on sternal size. We analyzed various combinations of explanatory variables on log_10_-transformed sternum centroid size (hereafter: “sternum size”) as the response variable, with log_10_-transformed cube-root body mass and locomotory variables as the explanatory variables. We fit models using the gls function in the nlme R package [116] using a phylogenetic correlation structure generated by the corPagel function in the ape package [117] with the value of Pagel’s lambda estimated during model fitting [115, 118]. Model comparison was performed using the Akaike Information Criterion corrected for small sample sizes (AICc) and AICc weights in the R package qpcR [119]. We also plotted the residuals of the regression line from the 'best' model (see results) against locomotory category to visualize the deviation of locomotory groups from allometric expectations.

#### Analysis of sternum shape

Applying phylogenetic comparative methods to high-dimensional data, such as 3D geometric morphometric data, has been a challenging endeavor until recently. When the effective number of variables (p*3 for *p* landmarks in 3D space) exceeds the number of observations (*N*), or species, an evolutionary trait covariance matrix is incomputable and thus the data is unanalyzable under classic variance-covariance matrix methods [120]. Several new approaches have been introduced in recent years, including dimensionality reduction into principal components (PCs), Euclidean distance-based pGLS/ANOVA [46], pairwise composite likelihoods [121], and more recently, a penalized likelihood approach to covariance matrices [122], each with their own benefits and drawbacks. Here, we apply the first two methods, for consistency and comparability with previous analyses of ecomorphology.

We visualized major aspects of sternal shape variation using phylogenetically informed principal component analysis (PCA) of shape (Procrustes coordinates), implemented via the gm.prcomp function in the geomorph package [110], to visualize the major axes of variation in sternal morphology. We also plotted the first four PC axes against body size to visualize the allometric relationship with these shape components (Additional file 1: Fig. S2).

For visualization purposes, datapoints in Figs. 3, 4, 5, and 6 are coloured according to only one locomotory mode, representing a simplified version of the locomotory categorization described above. We followed classification styles used in [9, 10, 68] to assign these generalized classifications and extended them based on their similarity to taxa included in this paper for ease of communication.

We tested the allometry and locomotor correlates of sternal shape using distance-based pGLS analysis of the Procrustes coordinates (shape). These analyses were implemented using the procD.pgls function in the geomorph package [110], using the Type II (hierarchical) sum of squares to account for phylogenetic clustering of locomotor variables [123]. This tested the hypothesized relationships of sternal shape to body mass and individual locomotory capabilities (Table 1). This method is desirable because it retains high statistical power when the effective number of variables (*3p*) exceeds the number of observations (*N*). This is achieved by analyzing pairwise distances between observations rather than estimating a covariance matrix [46]. We specifically evaluated models formulated as *shape ~ body mass + sternum size + locomotory mode 1 + locomotory mode 2*… to permit the assignment of multiple locomotory modes per species by coding them as 'present' or 'absent'. This allows us to explore the relationship between specific shape traits and each locomotory mode, rather than restricting each species to one mode only.

Various procD.pgls models were fit to test the influence of different combinations of explanatory variables—body mass, sternum size, and locomotory modes—on shape. Continuous-valued traits (body mass, centroid size) were log_10_-transformed prior to analysis. Model selection was then performed through stepwise regression, which chooses the best model based on the statistical significance of each variable after sequentially removing non-significant variables [124], as there is currently no adequate AIC-based model selection method for Procrustes shape data to our knowledge. Shape deformations associated with each explanatory variable in the model were computed and visualized to portray the independent effect of each explanatory variable in the model. This was done by warping a 3D sternum model used in the analysis, that of *Ramphastos ambiguus*, by the Procrustes shape variables in 3D shape space as hypothetical sternum renderings.

Shape allometry was assessed by performing a linear regression of the procD.pgls regression score coefficients—a variable that summarizes the shape changes predicted by the multivariate regression [125]—against body mass. Shape deformations for the first (25%) and third (75%) quartile of body mass were produced by warping a 3D sternum model by the Procrustes shape variables and plotted in 3D shape space, while keeping centroid size constantly proportional, to assess the influence of body mass alone on shape. Similarly, shape deformations for larger and smaller sternum centroid sizes were plotted by calculating the centroid size with the pGLS allometry equation and adding or subtracting (2 * standard error) from the allometry equation, while keeping body mass constant. The datasets generated and/or analyzed for this study are available in the Dryad [109] and MorphoSource [100] repositories.

## Supplementary Information

**Additional file 1.** Supplementary results, methods, Figures S1-S7, Tables S1-S2. Supplementary results – reporting on allometry regressions based on PC scores; Fig S1 – regression plots for allometry for first 4 PC axes. Fig S2 – point cloud shape deformations associated with body size and sternum size. Fig S3 - point cloud shape deformations associated with forelimb propulsion and aerial flight. Fig S4 - point cloud shape deformations associated with soaring, continuous flapping, and burst flight. Fig S5 – point cloud shape deformations associated with terrestriality and cursoriality. Fig S6 –point cloud shape deformations associated with foot-propelled diving compared with that of forelimb propulsion. Table S1 – regression statistics for all sternum size allometry models. Table S2 – regression statistics from allometry regressions based on PC axes. Supplementary methods - description the landmarking scheme; Fig S7 – depiction of landmark scheme.

## Data Availability

The datasets supporting the conclusions of this article and scripts for programs run are accessible in the Dryad repository doi: 10.5061/dryad.x3ffbg7jj [109]. All scan data is available on MorphoSource: www.morphosource.org/projects/000370001 [100].

## Abbreviations

ANOVA: Analysis of variance
pGLS: Phylogenetic generalized lease squares
AICc: Corrected Akaike Information Criterion
PCA: Principal component analysis
PC: Principal components

## Footnotes

1 Sterna in the primarily burst flying clade Galliformes have additional lateral processes, thought to be homologs of sternal ribs [28, 75–77], that flare outward from the lateral trabeculae and are distally expanded; these additional processes are not present in the other burst flying clades.
