# Supplementary material for: The relationship between sternum variation and mode of locomotion in birds
Source: BMC Biol. 2021 Aug 19;19:165. doi: 10.1186/s12915-021-01105-1 (PMC8377870; doi:10.1186/s12915-021-01105-1)
Supplement: Supplementary file 1 — Additional file 1. Supplementary results, methods, Figures S1-S7, Tables S1-S2. Supplementary results – reporting on allometry regressions based on PC scores; Fig S1 – regression plots for allometry for first 4 PC axes. Fig S2 – point cloud shape deformations associated with body size and sternum size. Fig S3 - point cloud shape deformations associated with forelimb propulsion and aerial flight. Fig S4 - point cloud shape deformations associated with soaring, continuous flapping, and burst flight. Fig S5 – point cloud shape deformations associated with terrestriality and cursoriality. Fig S6 –point cloud shape deformations associated with foot-propelled diving compared with that of forelimb propulsion. Table S1 – regression statistics for all sternum size allometry models. Table S2 – regression statistics from allometry regressions based on PC axes. Supplementary methods - description the landmarking scheme; Fig S7 – depiction of landmark scheme. [file 12915_2021_1105_MOESM1_ESM.pdf]

Additional File 1 for: Lowi-Merri et al. The relationship between sternum variation and mode of locomotion in birds in *BMC Biology*

Supplementary Results

Shape Allometry using PC Scores

PC axes were plotted against log cube root body mass to test for an allometric relationship between different sternum shape variables and body mass. PC1, defined by elongation of the sternum and lateral trabeculae, showed slight positive allometry with body mass (slope = 0.316, p = 0.000) (Fig. S2). This indicates that square-shaped sterna with an incurved caudal edge and a low, anteriorly angled keel occur more in larger-sized birds, while smaller birds typically possess sterna with a taller keel and a rounded caudal edge. PC2, defined by narrowing and widening of the sternum, showed a slight negative allometric relationship with body mass (slope = -0.155, p = 0.000). This indicates that larger birds tend to have more narrow sterna compared with smaller birds. PC3, defined by the height of the keel and shape of the caudal border, did not show a significant relationship with body mass (slope = 0.049, p = 0.1709), nor did PC4, which is defined by the angle of the keel (slope = 0.0365, p = 0.231). Therefore, keel height and angle are not correlated with body size, and these morphological features are likely influenced by some other ecological variable.

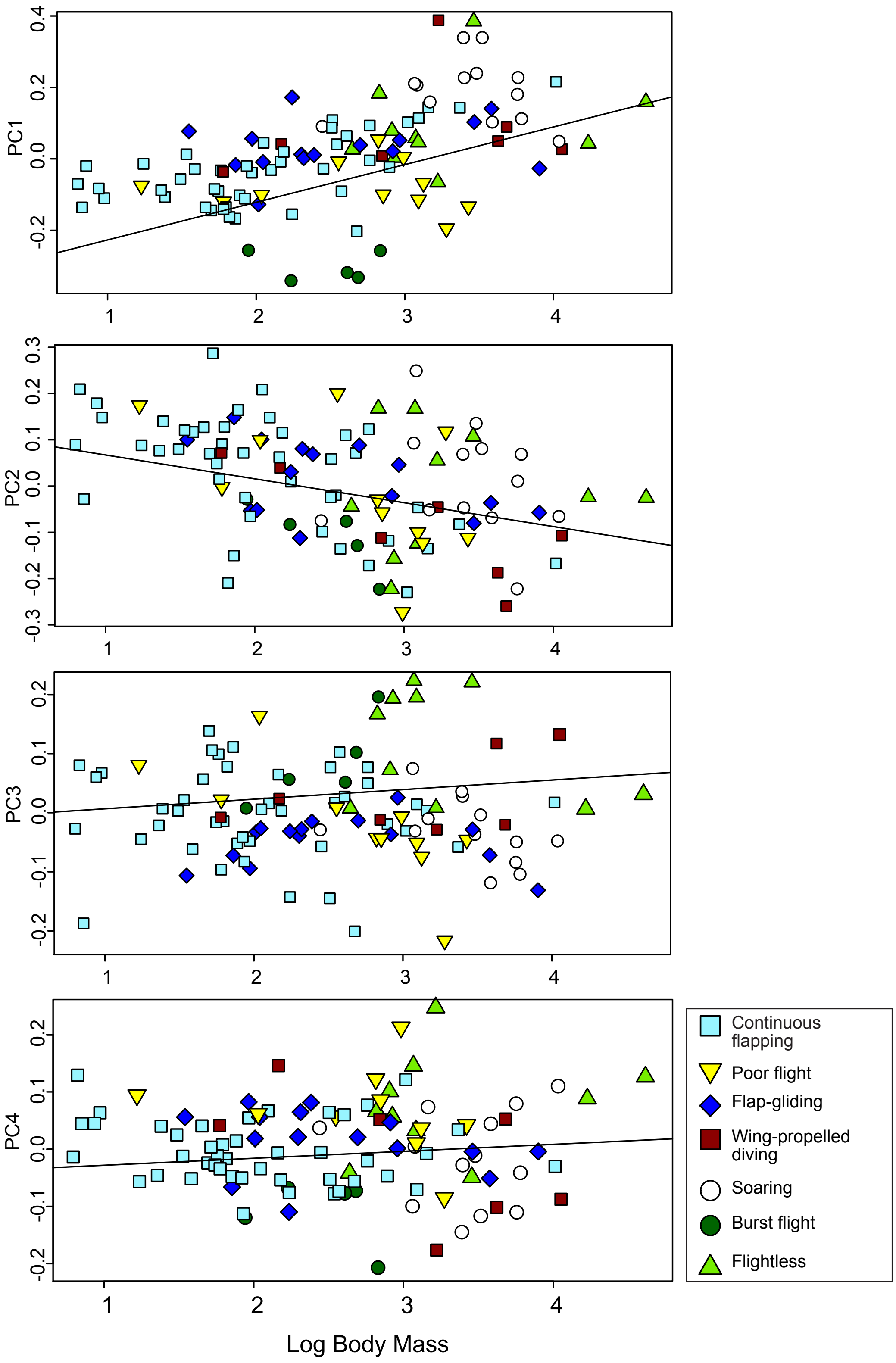

Figure S1: Regression plots for testing for allometry along the first 4 PC axes. Test statistics provided in Table S1.

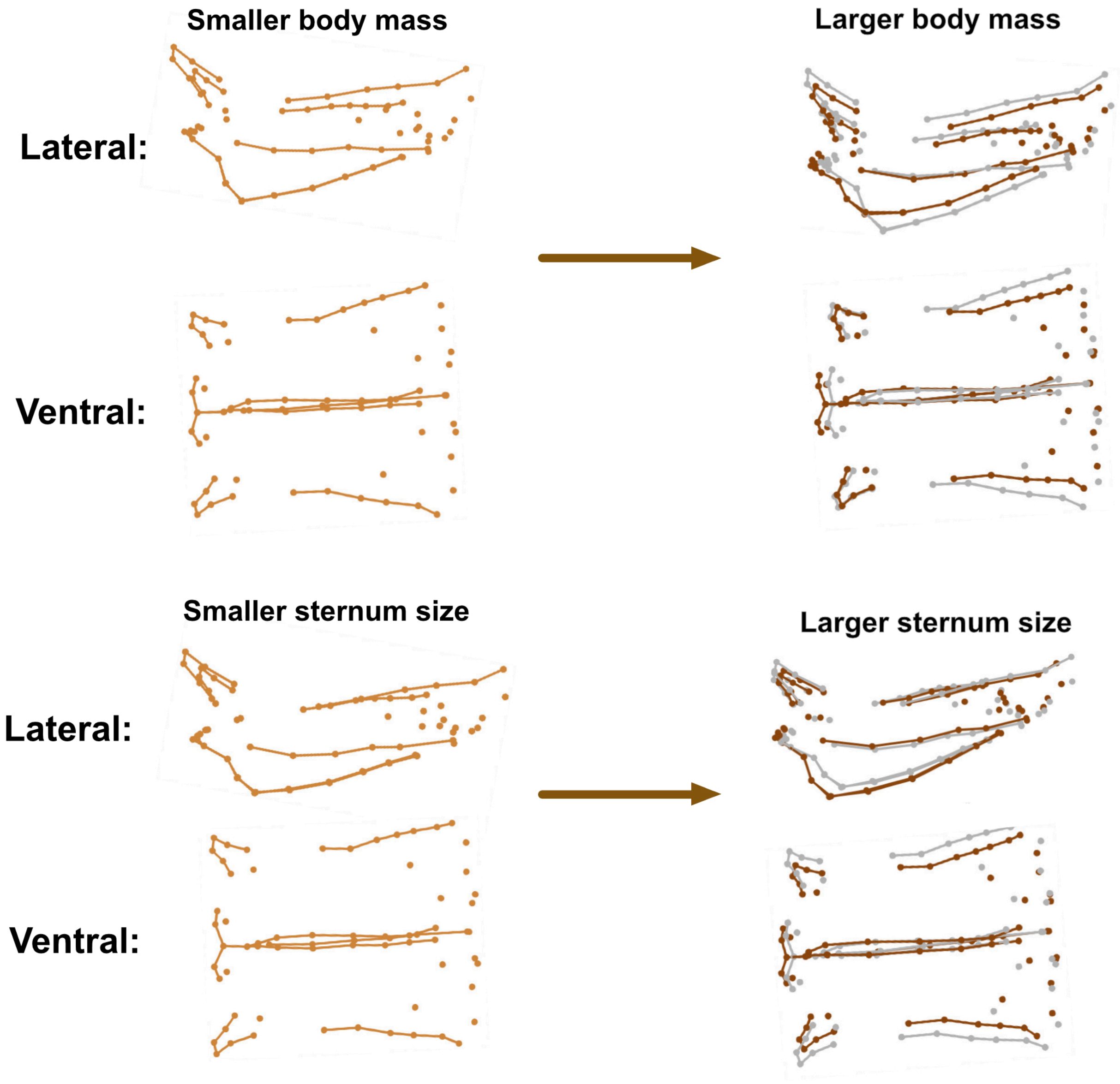

Figure S2: Sternal shape deformations showing the independent effect of sternum size and body size on sternum shape, showing smaller sizes in beige on the left and larger sizes in brown on the right. The grey shape overlain on the brown shape on the right (larger sternum size or body mass) represents the shape shown on the left side (smaller sternum size or body mass).

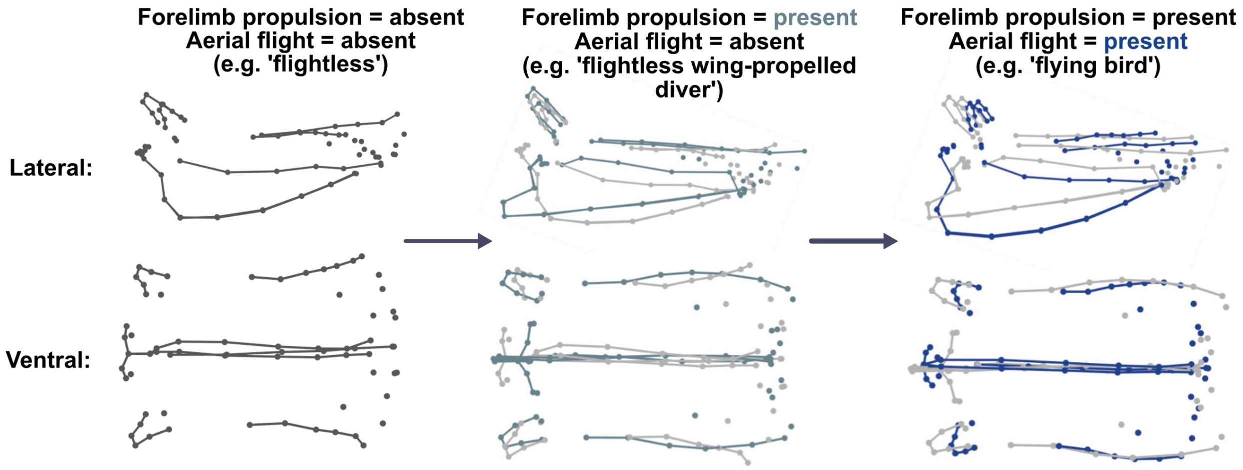

Figure S3: Shape deformations showing the independent effects of forelimb propulsion and aerial flight. The light grey shape overlain on top of the coloured shapes represents the coloured shape that occurred to the left in the series as a baseline for comparison. Forelimb propulsion is associated with a longer sternal body and lower, anteriorly projected keel; aerial flight is associated with a shorter sternal body and taller, rounded keel.

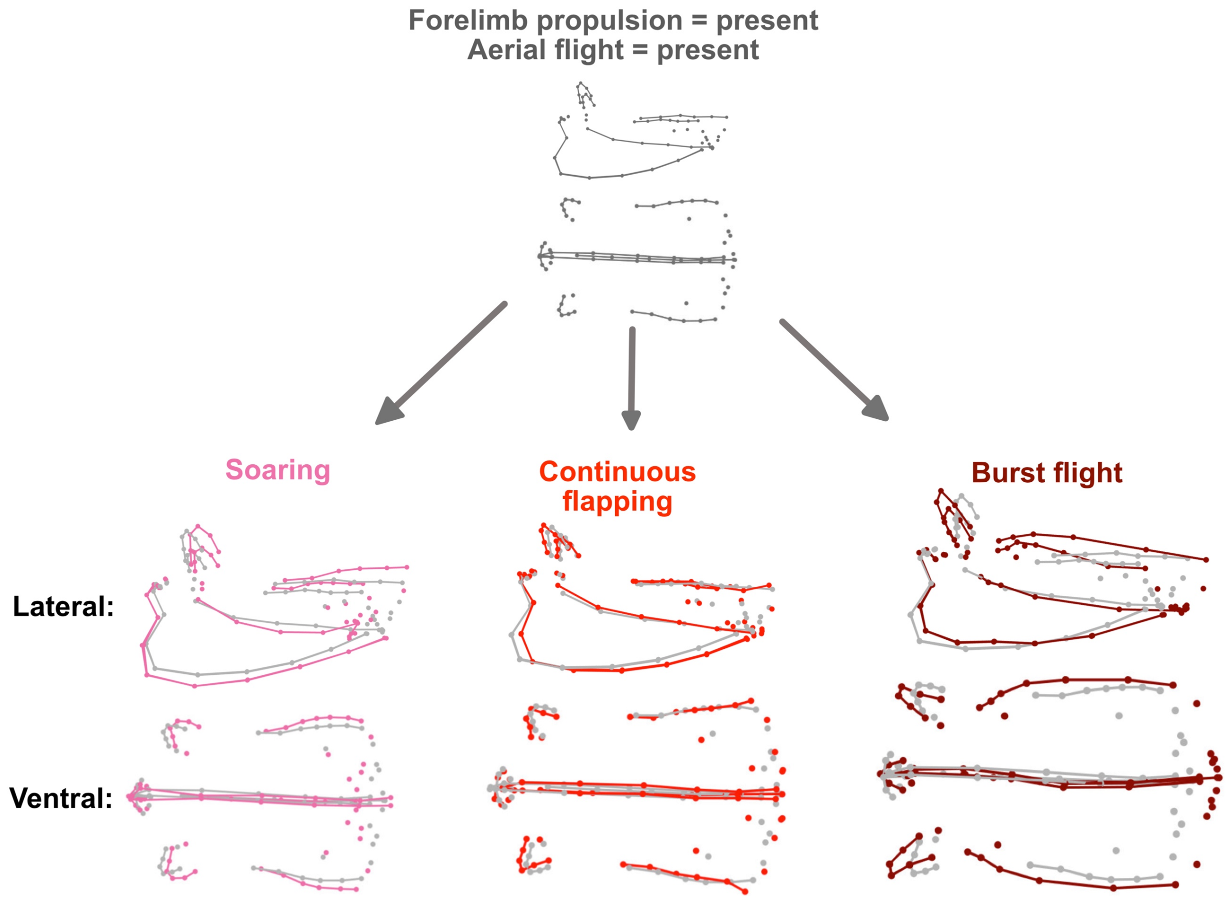

Figure S4: Shape deformations showing the independent effects of soaring flight, continuous flapping flight, and burst flight. The light grey shape overlain on top of the coloured shapes represents the shape shown at the top of the figure (showing the shape deformation for aerial flight) as a baseline for comparison.

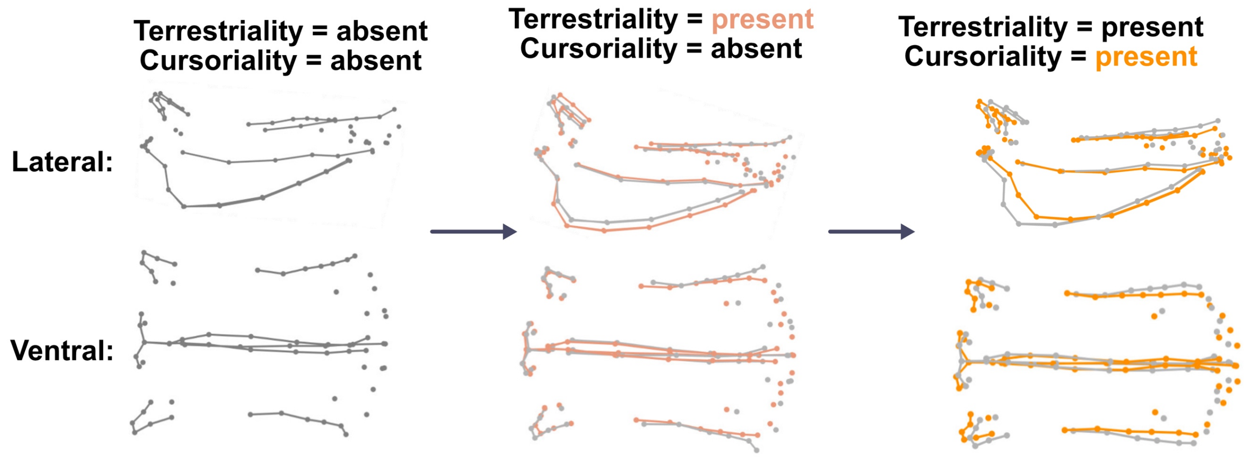

Figure S5: Shape deformations showing the independent effects of terrestriality and cursoriality. The light grey shape overlain on top of the coloured shapes represents the coloured shape occurring to the left in the series as a baseline for comparison.

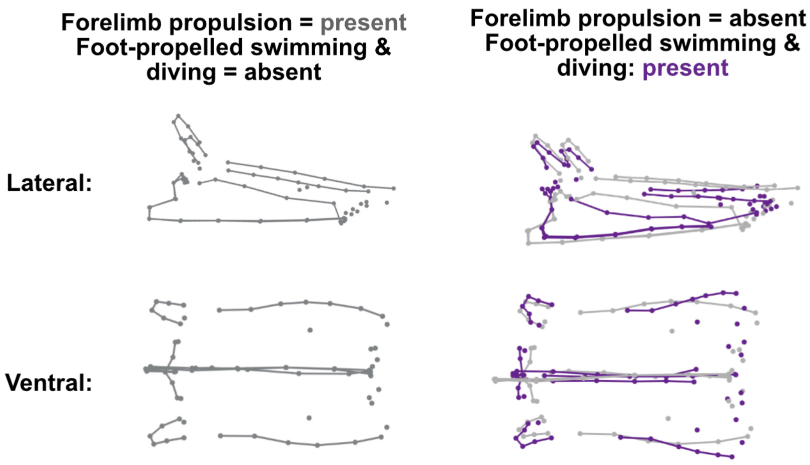

Figure S6: Shape deformations showing the independent effects of foot-propelled swimming and diving, with forelimb propulsion included for comparison. The light grey shape overlain on top of the coloured shapes represents the coloured shape occurring to the left in the series.

Table S1: Regression statistics for all remaining allometry regression models testing for allometry of sternum size. BM = body mass, size = sternum centroid size, FP = forelimb propulsion, AF = aerial flight, BF = burst flight, SO = soar, FPD = foot-propelled swimming/diving, WPD = wing-propelled diving, CURS = cursoriality, TERR = terrestriality, CF = continuous flapping. *N* = 105 for all analyses.

Models	AICc	AICc weights	R^2	Pagel's λ	Variable	Coefficient	Std Error	t-value	*p*-value
size ~ BM + BF	-217.26	1.19×10^{-6}	0.89	0.68	Intercept	1.44	0.048	29.86	0
					BM	0.99	0.033	30.57	0
					BF	0.089	0.044	2.008	0.047
size ~ BM + FP + AF + FPD + WPD + BF + SO + CURS	-208.10	1.2×10^{-8}	0.89	0.75	Intercept	1.32	0.052	25.53	0
					BM	1.015	0.033	30.69	0
					FP	0.20	0.054	3.70	0.0004
					AF	-0.077	0.05	-1.53	0.13
					FPD	0.03	0.024	1.26	0.21
					WPD	0.015	0.034	0.42	0.67
					BF	0.082	0.042	1.97	0.052
					SO	-0.0055	0.024	-0.23	0.82
					CURS	-0.002	0.019	-0.12	0.902
size ~ BM + FP + AF + BF + SO + CURS + TERR + CF	-203.76	1.4×10^{-9}	0.89	0.78	Intercept	1.32	0.053	24.70	0
					BM	1.023	0.035	29.14	0
					FP	0.19	0.045	4.28	0
					AF	-0.077	0.044	-1.75	0.083
					BF	0.079	0.043	1.86	0.066
					SO	-0.0042	0.024	-0.18	0.86
					CURS	-0.002	0.02	-0.11	0.91
					TERR	-0.0016	0.015	-0.11	0.91
					CF	0.0087	0.017	0.51	0.61

size ~ BM + FP + AF + FPD + WPD + BF + SO + CURS + TERR	-199.15	1.4×10^{-10}	0.88	0.74	Intercept	1.32	0.052	25.403	0
					BM	1.014	0.034	29.89	0
					FP	0.2004	0.055	3.67	0.0004
					AF	-0.078	0.051	-1.53	0.13
					FPD	0.032	0.024	1.30	0.20
					WPD	0.015	0.035	0.43	0.67
					BF	0.082	0.042	1.97	0.052
					SO	-0.006	0.024	-0.23	0.82
					CURS	-0.004	0.02	-0.18	0.86
					TERR	0.004	0.015	0.25	0.803
size ~ BM + FP + AF + FPD + WPD + BF + SO + CURS + TERR + CF	-190.50	1.8×10^{-12}	0.88	0.74	Intercept	1.31	0.053	24.71	0
					BM	1.02	0.036	28.61	0
					FP	0.20	0.055	3.61	0.0005
					AF	-0.081	0.052	-1.55	0.12
					FPD	0.031	0.025	1.24	0.22
					WPD	0.015	0.035	0.44	0.66
					BF	0.085	0.042	2.001	0.048
					SO	-0.003	0.026	-0.11	0.91
					CURS	-0.003	0.0201	-0.11	0.91
					TERR	0.004	0.015	0.28	0.78
					CF	0.007	0.017	0.39	0.70

Table S2: Regression statistics from linear regression between principal component (PC) axes and body mass

	N	Slope	95% CI	*p*	*P* from t-distr.
PC1 vs. log cube root body mass	105	0.32	-0.078 to -0.044	0	2.16×10^{-10}
PC2 vs. log cube root body mass	105	-0.16	-0.023 to -0.0058	0	0.001
PC3 vs. log cube root body mass	105	0.049	0.03 to 0.035	0.17	$< 2.2\times10^{-16}$
PC4 vs. log cube root body mass	105	0.037	-0.011 to -0.007	0.23	1.047×10^{-14}

Supplementary Methods

Descriptions for landmarks (LM) and sliding semi-landmarks (SL) in order of placement

LM1

Centre – The most anterior point of rostrum sterni. Sometimes this is confluent with the keel, sometimes it is separate.

SL1

Left – Side edge of rostrum sterni, landmarked from LM1 to LM2. Equivalent to SL2 on the right side.

LM2

Left – Corner between rostrum sterni and coracoid sulcus. Equivalent to LM3 on the right side.

SL2

Right – Side edge of rostrum sterni, landmarked from LM1 to LM3. Equivalent to SL1 on the left side.

LM3

Right – Corner between rostrum sterni and coracoid sulcus. Equivalent to LM2 on the left side.

LM4

Left – Most medial point of the left coracoid sulcus. Equivalent to LM6 on the right side.

LM5

Left – Most distal point of the left coracoid sulcus. Equivalent to LM7 on the right side.

LM6

Right – Most medial point of the right coracoid sulcus. Equivalent to LM4 on the left side.

LM7

Right – Most distal point of the right coracoid sulcus. Equivalent to LM5 on the left side.

LM8

Left – Corner/inflection point between main sternum body and left craniolateral notch. Equivalent to LM9 on the right side.

SL3

Left – Perimeter of left craniolateral process, landmarked from LM8 until LM10. Equivalent to SL4 on the right side.

LM9

Right – Corner/inflection point between main sternum body and right craniolateral notch. Equivalent to LM8 on the left side.

SL4

Right – Perimeter of right craniolateral process, landmarked from LM9 until LM12. Equivalent to SL3 on the left side.

LM10

Left – Anterior of anterior-most rib facet on the left side. Equivalent to LM12 on the right side.

LM11

Left – Posterior of posterior-most rib facet on the left side. Equivalent to LM13 on the right side.

LM12

Right – Anterior of anterior-most rib facet on the right side. Equivalent to LM10 on the left side.

LM13

Right – Posterior of posterior-most rib facet on the right side. Equivalent to LM11 on the left side.

SL5

Left – Lateral edge of sternum, landmarked from L11 to LM15. Equivalent to SL6 on right side.

SL6

Right – Lateral edge of sternum, landmarked from L13 to LM22. Equivalent to SL5 on left side.

LM14

Centre – Midpoint of sternum on posterior edge in ventral view.

LM15

Left – Caudolateral distal corner of sternum, or of lateral trabecula when present. Equivalent to L22 on right side.

LM16

Left – Medial corner of lateral trabecula when present. If a lateral trabecula is absent, place a series of semi-landmarks between the last landmark that was placed (eg. LM15) and LM14 and skip the remaining landmarks of the posterior border on the left side (LM17-21), as they are later estimated as equidistant midpoints derived from this semi-landmark series. If a fenestra is present, it should be treated as a lateral trabecula that has fused; this landmark should be placed along the posterior border directly posterior to the midpoint of the fenestra. Equivalent to LM23 on the right side.

LM17

Left – Most anterior point of the most distal sternal notch (if two notches are present; if only one notch is present, this landmark denotes it). If this notch is absent, place a series of semi-landmarks between the last landmark that was placed and LM14, and skip the remaining landmarks of the posterior border on the left side (LM18-21), as they are later estimated as equidistant midpoints derived from this semi-landmark series. If a fenestra is present, it should

be treated as a lateral trabecula that has fused; this landmark should be placed on the most anterior point of the fenestra. Equivalent to LM24 on the right side.

LM18

Left – Distal corner of intermediate trabecula or central sternum body (xiphoid process). If an intermediate trabecula is absent, place LM18 on the distal corner of the xiphoid process, place a series of semi-landmarks between LM18 and LM14, and skip the remaining landmarks of the posterior border on the left side (LM19-21), as they are later estimated as equidistant midpoints derived from this semi-landmark series. If a fenestra is present, it should be treated as an intermediate trabecula that has fused; this landmark should be placed along the posterior border directly posterior to the midpoint of the fenestra (in the same spot as the most recent landmark placed on the posterior border, LM16). Equivalent to LM25 on the right side.

LM19

Left – Medial corner of intermediate trabecula. If an intermediate trabecula is absent, place a series of semi-landmarks between the last landmark that was placed and LM14, skip the remaining landmarks of the posterior border on the left side (LM20-21), as they are later estimated as equidistant midpoints derived from this semi-landmark series. If a fenestra is present, it should be treated as an intermediate trabecula that has fused; this landmark should be placed along the posterior border directly posterior to the midpoint of the fenestra. Equivalent to LM26 on the right side.

LM20

Left – Most anterior point of medial sternum notch, if two notches are present. If this notch is absent, place a series of semi-landmarks between the last landmark that was placed and LM14, skip the remaining landmarks of the posterior border on the left side (L21) as they are later estimated as equidistant midpoints derived from this semi-landmark series. If a fenestra is present, it should be treated as an intermediate trabecula that has fused; this landmark should be placed on the most anterior point of the fenestra. Equivalent to LM27 on the right side.

LM21

Left – Distal corner of xiphoid process. If this point does not exist on the posterior border (eg. the sternum does not have both a lateral trabecula and an intermediate trabecula), place a series of semi-landmarks between the last landmark that was placed and LM14, skip this landmark, as it will later be estimated as the equidistant midpoint derived from this semi-landmark series. If a fenestra is present, it should be treated as an intermediate trabecula that has fused; this landmark should be placed along the posterior border directly posterior to the midpoint of the fenestra (in the same spot as the most recent landmark placed on the posterior border, LM19). Equivalent to LM28 on the right side.

LM22

Right – Caudolateral distal corner of sternum, or of lateral trabecula, when present. Equivalent to L15 on left side.

LM23

Right – Medial corner of lateral trabecula when present. If a lateral trabecula is absent, place a series of semi-landmarks between the last landmark that was placed (eg. LM22) and LM14 and skip the remaining landmarks of the posterior border on the right side (LM24-28), as they are later estimated as equidistant midpoints derived from this semi-landmark series. If a fenestra is present, it should be treated as a lateral trabecula that has fused; this landmark should be placed along the posterior border directly posterior to the midpoint of the fenestra. Equivalent to LM16 on the left side.

LM24

Right – Most anterior point of the most distal sternal notch (if two notches are present; if only one notch is present, this landmark denotes it). If this notch is absent, place a series of semi-landmarks between the last landmark that was placed and LM14, and skip the remaining landmarks of the posterior border on the right side (LM25-28), as they are later estimated as equidistant midpoints derived from this semi-landmark series. If a fenestra is present, it should be treated as a lateral trabecula that has fused; this landmark should be placed on the most anterior point of the fenestra. Equivalent to LM17 on the left side.

LM25

Right – Distal corner of intermediate trabecula or central sternum body (xiphoid process). If an intermediate trabecula is absent, place LM25 on the distal corner of the xiphoid process, place a series of semi-landmarks between the last landmark that was placed and LM14, and skip the remaining landmarks of the posterior border on the right side (LM26-28), as they are later estimated as equidistant midpoints derived from this semi-landmark series. If a fenestra is present, it should be treated as an intermediate trabecula that has fused; this landmark should be placed along the posterior border directly posterior to the midpoint of the fenestra (in the same spot as the most recent landmark placed on the posterior border, LM23). Equivalent to LM18 on the left side.

LM26

Right – Medial corner of intermediate trabecula. If an intermediate trabecula is absent, place a series of semi-landmarks between the last landmark that was placed and LM14, skip the remaining landmarks of the posterior border on the right side (LM27-28), as they are later estimated as equidistant midpoints derived from this semi-landmark series. If a fenestra is present, it should be treated as an intermediate trabecula that has fused; this landmark should be placed along the posterior border directly posterior to the midpoint of the fenestra. Equivalent to LM19 on the left side.

LM27

Right – Most anterior point of medial sternum notch, if two notches are present. If this notch is absent, place a series of semi-landmarks between the last landmark that was placed and LM14, skip the remaining landmarks of the posterior border on the right side (L28) as they are later estimated as equidistant midpoints derived from this semi-landmark series. If a fenestra is present, it should be treated as an intermediate trabecula that has fused; this landmark should be placed on the most anterior point of the fenestra. Equivalent to LM20 on the left side.

LM28

Right – Distal corner of xiphoid process. If this point does not exist on the posterior border (eg. the sternum does not have both a lateral trabecula and an intermediate trabecula), place a series

of semi-landmarks between the last landmark that was placed and LM14, skip this landmark, as it will later be estimated as the equidistant midpoint derived from this semi-landmark series. If a fenestra is present, it should be treated as an intermediate trabecula that has fused; this landmark should be placed along the posterior border directly posterior to the midpoint of the fenestra (in the same spot as the most recent landmark placed on the posterior border, LM26). Equivalent to LM21 on the left side.

LM29

Left – Most posterior corner of sternal keel. Equivalent to LM30 on the right side.

SL7

Left – Perimeter of sternal keel, landmarked from LM29 to LM1. Equivalent to SL8 on right side.

LM30

Right – Most posterior corner of sternal keel. Equivalent to LM29 on the left side.

SL8

Right – Perimeter of sternal keel, landmarked from LM30 to LM1. Equivalent to SL7 on left side.

LM31

Centre – Midpoint of sternum on posterior edge in dorsal view.

SL9

Centre – Dorsal midline, landmarked from LM31 to LM32.

LM32

Centre – Dorsal midpoint, just before dorsal surface slopes anterodorsally.

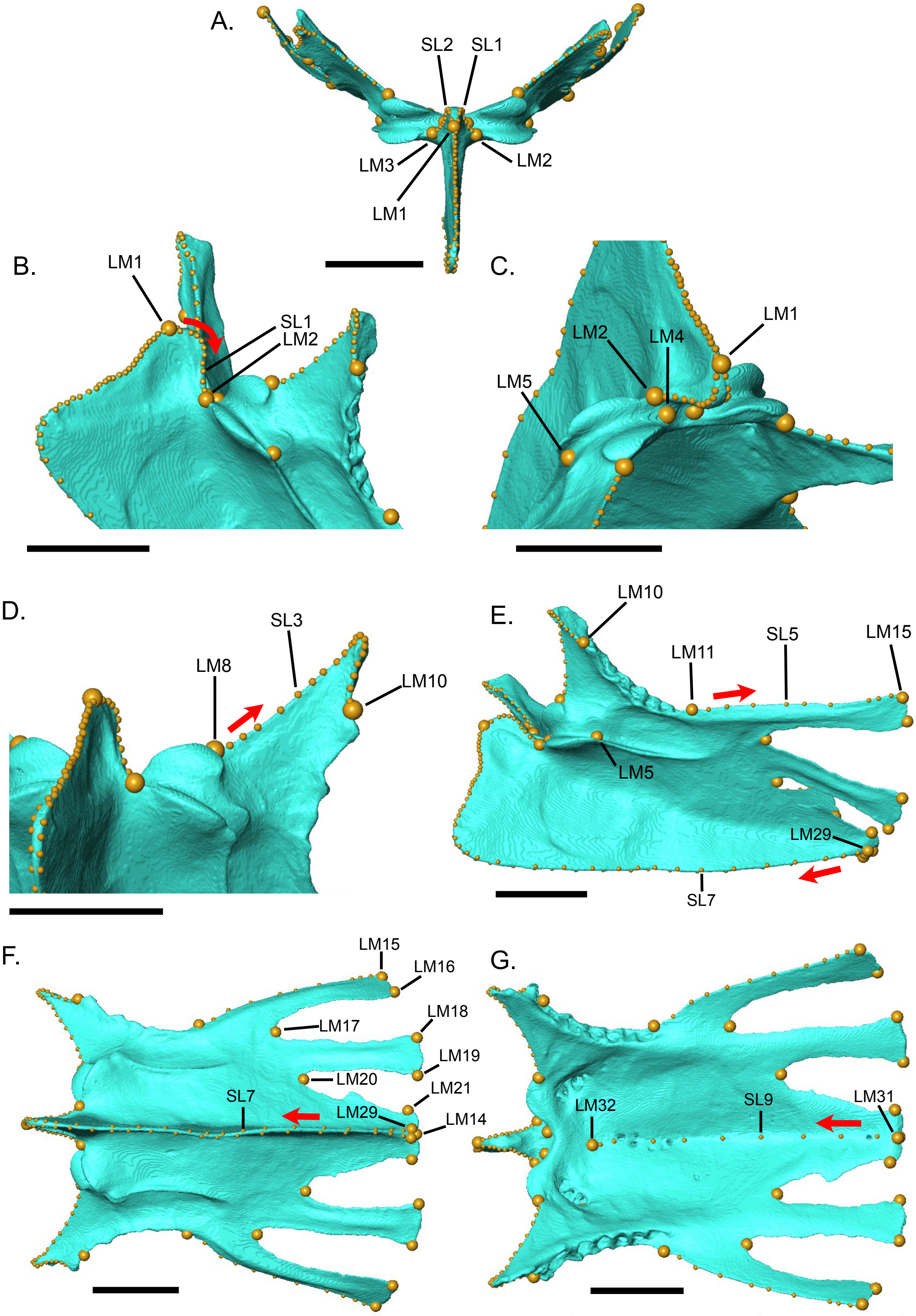

A.
SL2
SL1
LM3
LM2
LM1
B.
LM1
SL1
LM2
C.
LM1
LM2
LM4
LM5
D.
SL3
LM8
LM10
E.
LM10
LM11
SL5
LM15
LM5
LM29
SL7
F.
LM15
LM16
LM17
LM18
LM19
LM20
LM21
SL7
LM29
LM14
G.
LM32
SL9
LM31

Figure S7: Sternum landmarks (LM) and semi-landmark (SL) series shown on the sternum of *Ramphastos ambiguus* (NHMUK S2002.21.2). LM are noted by the larger yellow spheres, and SL by the smaller yellow spheres. Red arrows indicate the direction in which the semilandmarks are placed. A. Sternum in anterior view. LM1-3 and SL1-2 are shown. B. Anterior half of sternum shown in lateral view. LM1-2 and SL1 are shown. C. Anterior half sternum shown in dorsolateral view to focus on coracoid sulcus. LM1-5 are shown. D. Anterior half sternum shown in ventrolateral view to focus on the craniolateral process. LM8, 10 and SL3 are shown. E. Sternum in left lateral view. LM5, 10, 11, 15, 29, and SL5 and 7 are shown. F. Sternum in ventral view. LM14-21, 29 and SL7 are shown. G. Sternum in dorsal view. LM31-32 and SL9 are shown. Landmarks which occur on both the left and right side (labelled 'Left' or 'Right' in the description above) are labelled only on the left side, except in A. Equivalent landmarks on the right side are described above. Scale bars: 10 mm.

Simplified flight styles in BM_flightsimp.csv: BF = burst flight; CF = continuous flapping; FG = flap-gliding; FL = flightless; PF = poor flight; SO = soaring; WD = wing-propelled diving
